# Accounting for misclassified and unknown cause of death data in vital registration systems for estimating trends in HIV mortality

**DOI:** 10.1002/jia2.25791

**Published:** 2021-09-21

**Authors:** Hmwe H. Kyu, Deepa Jahagirdar, Matthew Cunningham, Magdalene Walters, Edmond Brewer, Amanda Novotney, Eve Wool, Ilse Dippennar, Fablina Sharara, Chieh Han, Shelly Balassyano, Greg Bertolacci, Christopher J. L. Murray, Mohsen Naghavi

**Affiliations:** ^1^ Institute for Health Metrics and Evaluation Seattle Washington USA; ^2^ Department of Health Metrics Sciences University of Washington Seattle Washington USA

**Keywords:** cause of death, garbage coding, HIV mortality, misclassification

## Abstract

**Introduction:**

Misclassification of HIV deaths can substantially diminish the usefulness of cause of death data for decision‐making. In this study, we describe the methods developed by the Global Burden of Disease Study to account for the misclassified cause of death data from vital registration systems for estimating HIV mortality in 132 countries and territories.

**Methods:**

The cause of death data were obtained from the World Health Organization Mortality Database and official country‐specific mortality databases. We implemented two steps to adjust the raw cause of death data: (1) redistributing garbage codes to underlying causes of death, including HIV/AIDS by applying methods, such as analysis of multiple cause data and proportional redistribution, and (2) reassigning HIV deaths misclassified as other causes to HIV/AIDS by examining the age patterns of underlying causes in location and years with and without HIV epidemics.

**Results:**

In 132 countries, during the period from 1990 to 2018, 1,848,761 deaths were reported as caused by HIV/AIDS. After garbage code redistribution in these 132 countries, this number increased to 4,165,015 deaths. An additional 1,944,291 deaths were added through correction of HIV deaths misclassified as other causes in 44 countries. The proportion of HIV deaths derived from garbage code redistribution decreased over time, from 0.4 in 1990 to 0.1 in 2018. The proportion of deaths derived from HIV misclassification correction peaked at 0.4 in 2006 and declined afterwards to 0.08 in 2018. The greatest contributors to garbage code redistribution were “immunodeficiency antibody” (ICD 9: 279‐279.1; ICD 10: D80‐D80.9) and “immunodeficiency other” (ICD 9: 279, 279.5‐279.9; ICD 10: D83‐D84.9, D89, D89.8‐D89.9), which together contributed 77% of all redistributed deaths at their peak in 1995. Respiratory tuberculosis (ICD 9: 010–012.9; ICD 10: A10‐A14, A15‐A16.9) contributed the greatest proportion of all HIV misclassified deaths (25–62% per year) over the most years.

**Conclusions:**

Correcting for miscoding and misclassification of cause of death data can enhance the utility of the data for analyzing trends in HIV mortality and tracking progress toward the Sustainable Development Goal targets.

## 1 INTRODUCTION

Accurate measurement of mortality and causes of death is an essential basis of policy planning and prioritizing interventions. To facilitate systematic recording and comparison of morbidity and mortality data, the International Classification of Diseases (ICD) is used globally as the standard diagnostic classification tool [1]. The ICD is periodically revised to reflect new scientific knowledge of health and disease [2]. HIV/AIDS is a relatively recent addition to the ICD with changes in coding between the 9th and 10th revisions of the ICD [1, 3]. However, despite the availability of ICD coding rules on systematic selection of the underlying cause of death [1, 4], misclassification of deaths in ICD‐coded vital registration data is common [5]. For instance, ICD codes for immediate causes of death (e.g. respiratory failure and cardiac arrest) or intermediate causes of death (e.g. sepsis and heart failure) are frequently classified as the underlying cause of death. ICD codes for immediate causes, intermediate causes or ill‐defined causes of death (e.g. sequelae of unspecified infectious and parasitic diseases) are often referred to as “garbage codes” [5] as they do not represent the underlying cause of death that triggered the sequence of events leading to death.

The use of garbage codes varies substantially across countries and over time, resulting in incomparability of the cause‐of‐death data. Certification and coding of certain causes, such as HIV/AIDS, is especially challenging because of multiple factors, including the substantial stigma associated with the disease, confidentiality‐related concerns and the similarity of numerous signs and symptoms of HIV/AIDS with that of other diseases [6]. Additionally, people with HIV infection are vulnerable to opportunistic infections (e.g. cryptococcal meningitis and cerebral toxoplasmosis) [7], co‐infections (e.g. HIV‐tuberculosis co‐infection and HIV‐hepatitis B virus co‐infection) [8], certain malignant neoplasms (e.g. Kaposi sarcoma and non‐Hodgkin lymphoma) [9] and other comorbid conditions (e.g. endocrine disorders) [10], and these conditions are often incorrectly assigned as the underlying cause of death [11]. Four types of misclassification of HIV deaths can occur: (1) incorrectly assigning intermediate causes or ill‐defined causes as the underlying cause of death; (2) assignment of HIV deaths to relevant garbage codes, such as unspecified immunodeficiency; (3) allocation of HIV deaths to diseases that can mimic HIV infection (e.g. inflammatory bowel disease and some skin diseases); and (4) misassigning HIV deaths to other underlying causes of death, such as tuberculosis and meningitis.

To enable a meaningful comparison of cause‐of‐death data, it is essential to reallocate deaths assigned to garbage codes or misclassified as other causes to the target underlying causes. Measuring progress towards attainment of the Sustainable Development Goal target 3 to end the HIV/AIDS epidemic by 2030 requires a reduction in new HIV infections and HIV/AIDS‐related deaths by 90% between 2010 and 2030 [12, 13]. Robust and valid data on causes of death are thus crucial for tracking the success of programs aimed at reducing HIV/AIDS mortality. A comprehensive framework for adjusting vital registration data to enhance the accuracy and comparability of cause‐of‐death data for the Global Burden of Disease study (GBD) has been published elsewhere [14]. The objective of this paper is to describe the methods used to account for the misclassified cause of death data in 132 countries and territories for estimating trends in HIV mortality.

## 2 METHODS

### 2.1 Overview

The GBD provides a standardized approach to addressing the challenges of measuring causes of death, including variability in the completeness of vital registration data, deaths attributed to garbage codes and misclassification of HIV deaths [11, 14, 15]. The GBD geographic hierarchy comprises 204 countries and territories grouped within 21 regions (based on epidemiological commonality and geographic closeness) and seven super‐regions (groupings of GBD regions based on cause‐of‐death patterns) (Appendix S1). GBD 2019 evaluated the overall quality of the cause‐of‐death data from each country based on factors, including completeness, garbage coding and time periods covered, and gave a quality rating of 0 stars (the poorest) to 5 stars (the best) (Appendix S1) [11]. The detailed methods used to assess the quality of the data and adjust vital registration completeness have been published elsewhere [11, 15]. Here, we describe the methods used for correcting misclassified cause of death data from vital registration systems in 132 countries and territories for estimating HIV mortality.

### 2.2 Input data

Every year, member states report to the World Health Organization (WHO) the number of deaths by sex, age group and cause of death registered through national civil registration. WHO harmonizes these data in the WHO Mortality Database and publicly release data for research purposes. Additional cause‐of‐death data were obtained from official country‐specific mortality databases. Country‐specific data sources are provided Appendix S1. We adjusted all the raw data in any format for garbage coding and HIV misclassification by (1) redistributing garbage codes to underlying causes of death including HIV/AIDS, and (2) reassigning misclassified HIV deaths to HIV. These adjustments are implemented by age, sex, year and location using the methods described below.

### 2.3 Redistribution of HIV/AIDS‐related garbage codes

To determine the proportion of HIV/AIDS‐related garbage codes (Table 1) to be reassigned to HIV/AIDS and other underlying causes of death, we first generated target proportions for each garbage group by 5‐year time interval and sex for the following age categories: under 1 month, 1–59 months, 5–19 years, 20–49 years, 50–59 years, 60–69 years, 70–79 years and 80+ years. The assignment of deaths to HIV or other underlying causes is based on the level of regional increase in the mortality rate for ICD codes shown in Table 1 in each group relative to the rates observed during the period from 1980 to 1984. We assumed that an increase of more than 5% is HIV/AIDS‐related, and reassigned the proportion of those excess deaths over 5% to HIV/AIDS. For an increase of 5% or less, the excess deaths were assigned to other underlying causes of death. The 5% cut‐off was arbitrarily chosen based on available data on the pattern of age‐specific mortality rates.

**Table 1:** HIV/AIDS‐related garbage code redistribution packages

Package name	ICD9 codes	ICD10 codes
Actinomycosis	39, 39.0, 39.1, 39.2, 39.3, 39.4, 39.6, 39.8, 39.9, 113, 113.2, 113.4, 113.5, 113.6	A42, A42.0, A42.1, A42.2, A42.7, A42.8, A42.81, A42.82, A42.89, A42.9
Bartonellosis	88.0, 88.2, 88.3, 88.5, 88.7	A44, A44.0, A44.1, A44.8, A44.9
Urogenital candidiasis	112.1, 112.2	B37.3, B37.4, B37.41, B37.42, B37.49
Candidiasis	112, 112.0, 112.3, 112.4, 112.5, 112.6, 112.8, 112.81, 112.82, 112.83, 112.84, 112.85, 112.89, 112.9	B37, B37.0, B37.1, B37.2, B37.5, B37.6, B37.7, B37.8, B37.81, B37.82, B37.83, B37.84, B37.89, B37.9
Coccidioidomycosis	114, 114.0, 114.1, 114.2, 114.3, 114.4, 114.5, 114.6, 114.9	B38, B38.0, B38.1, B38.2, B38.3, B38.4, B38.7, B38.8, B38.81, B38.89, B38.9
Histoplasmosis	115, 115.0, 115.00, 115.01, 115.02, 115.03, 115.04, 115.05, 115.09, 115.1, 115.10, 115.11, 115.12, 115.13, 115.14, 115.15, 115.19, 115.2, 115.3, 115.4, 115.5, 115.9, 115.90, 115.91, 115.92, 115.93, 115.94, 115.95, 115.99	B39, B39.0, B39.1, B39.2, B39.3, B39.4, B39.5, B39.9
Blastomycosis	116, 116.0, 116.2, 116.3, 116.4, 116.5, 116.6, 116.9	B40, B40.0, B40.1, B40.2, B40.3, B40.7, B40.8, B40.81, B40.89, B40.9
Paracoccidioidomycosis	116.1	B41, B41.0, B41.7, B41.8, B41.9
Sporotrichosis and chromomycosis	117.1	B42, B42.0, B42.1, B42.7, B42.8, B42.81, B42.82, B42.89, B42.9, B43, B43.0, B43.1, B43.2, B43.8, B43.9
Aspergillosis	117.3	B44, B44.0, B44.1, B44.2, B44.7, B44.8, B44.81, B44.89, B44.9
Zygomycosis	117.7	B46, B46.0, B46.1, B46.2, B46.3, B46.4, B46.5, B46.8, B46.9
Toxoplasmosis	130, 130.0, 130.1, 130.2, 130.3, 130.4, 130.5, 130.6, 130.7, 130.8, 130.9	B58, B58.0, B58.00, B58.01, B58.09, B58.1, B58.2, B58.3, B58.8, B58.81, B58.82, B58.83, B58.89, B58.9
Pneumocystosis, psorospermiasis and sarcosporidiosis	136.3, 136.4, 136.5	B59, B59.0, B59.9
Cryptococcosis	117.5	B45, B45.0, B45.1, B45.2, B45.3, B45.7, B45.8, B45.9
Chromoblastomycosis/nocardiosis	117.2	A43, A43.0, A43.1, A43.8, A43.9
Mycoses/unspecified mycosis	117, 117.0, 117.4, 117.6, 117.8, 117.9, 118, 118.0, 118.1, 118.2, 118.3, 118.4, 118.5, 118.6, 118.9	B49, B49.5, B49.9
Cutaneous leishmaniasis	85.1, 85.2, 85.3, 85.4, 85.5	B55.1, B55.2
Other mycobacterial infection	31, 31.0, 31.2, 31.8, 31.9	A31, A31.0, A31.8, A31.9
Mycobacterial skin infection	31.1	A31.1, A31.2
Immunodeficiency – antibody	279.0, 279.00, 279.01, 279.02, 279.03, 279.04, 279.05, 279.06, 279.09, 279.1	D80, D80.0, D80.1, D80.2, D80.3, D80.4, D80.5, D80.6, D80.7, D80.8, D80.9
Immunodeficiency – WBC	279.10, 279.11, 279.12, 279.13, 279.19, 279.2, 279.3, 279.4, 279.41, 279.49	D81, D81.0, D81.1, D81.2, D81.3, D81.4, D81.5, D81.6, D81.7, D81.8, D81.81, D81.810, D81.818, D81.819, D81.89, D81.9, D82, D82.0, D82.1, D82.2, D82.3, D82.4, D82.8, D82.9
Immunodeficiency – other	279, 279.5, 279.50, 279.51, 279.52, 279.53, 279.6, 279.8, 279.9	D83, D83.0, D83.1, D83.2, D83.8, D83.9, D84, D84.0, D84.1, D84.8, D84.9, D89.8, D89.81, D89.810, D89.811, D89.812, D89.813, D89.82, D89.89, D89.9
Kaposi's sarcoma	176, 176.0, 176.1, 176.2, 176.3, 176.4, 176.5, 176.8, 176.9	C46, C46.0, C46.1, C46.2, C46.3, C46.4, C46.5, C46.50, C46.51, C46.52, C46.6, C46.7, C46.8, C46.9

### 2.4 Redistribution of other garbage codes

We used two methods to redistribute other garbage codes (Appendix S1) to HIV/AIDS. For ill‐defined causes (Appendix S1), proportional redistribution was used. We generated the proportions based on the distribution of the target ICD codes in the data by age, sex, location and year. These target codes were defined based on multiple cause data, pathology and evidence from the literature. Deaths from the ill‐defined causes were then split proportionally over all target underlying causes, including HIV/AIDS. For HIV deaths assigned to intermediate causes, such as sepsis, we determined the fraction of deaths due to HIV/AIDS based on our analysis of the multiple causes of death data. Multiple causes of death data include a combination of an underlying cause of death and other causes, such as immediate and intermediate causes, that were included in the series of events leading to death. Analyzing multiple causes of death data can provide insight into identifying the true underlying cause of death in data from other sources where the underlying cause is incorrectly assigned to a garbage code.

Multiple causes of death data from the United States, Mexico, Brazil, Taiwan, Italy and Colombia were used to inform the redistribution of the following intermediate causes to HIV/AIDS and other target underlying causes: acute renal failure; acute respiratory failure; cachexia; chronic respiratory failure; empyema; fluid, electrolyte and acid‐base disorders; hepatic failure; unspecified central nervous system disorders; osteomyelitis; peritonitis; pneumonitis; pulmonary embolism; sepsis (excluding maternal and neonatal sepsis); and shock, cardiac arrest and coma. We first ran a generalized linear model to estimate the fraction of deaths related to intermediate causes for each underlying cause as a function of covariates (Appendix S1). Next, we calculate the number of deaths related to intermediate causes by multiplying the estimated fractions by the GBD 2019 cause‐specific death estimates [11]. We then compute the “intermediate cause fraction” for each underlying cause by dividing the number of intermediate cause‐related deaths by total intermediate cause‐related deaths by location, year, age and sex. These intermediate cause fractions were used to inform the redistribution of the intermediate‐cause‐related deaths to HIV/AIDS and other underlying causes.

Below is an example for sepsis, where a,s, l, y, c represent a given age group, sex, location, year and underlying cause of death:

(1) $$\mathrm{logit}\;\left(\mathrm{seps}\mathrm{is}\;\mathrm{frac}\mathrm{tion}\right)=\beta_{0}+\beta_{1}\mathrm{HAQ}+\beta_{2}\mathrm{sex}+Y_{\mathrm{cause}}+\varepsilon$$

(2) $$sepsis\;deaths_{a,s,l,y,c}=sepsis\;fraction_{a,s,l,y,c}*\;\;cause\;specific\;deaths_{a,s,l,y,c}$$

(3) $$total\;sepsis\;deaths_{a,s,l,y}=\sum_{c}sepsis\;deaths_{a,s,l,y,c}$$

(4) $$fraction\;of\;sepsis\;to\;redistribute_{a,s,l,y}=\frac{sepsis\;deaths_{a,s,l,y,c}}{total\;sepsis\;deaths_{a,s,l,y}},$$

where *sepsis fraction* is the proportion of deaths related to sepsis, $\beta_{0}$ is the global intercept, *HAQ* is the Healthcare Access and Quality Index (a measure on a scale of 0–100 created based on 32 causes for which mortality is amenable to healthcare) [16], *sex* is an indicator variable on sex, *Y_cause_* is the random effect on the underlying cause of death, such as HIV, and causespecificdeaths is GBD 2019 estimated cause‐specific deaths. Separate generalized linear models were run for each age group. Due to the limited availability of data, we applied the country‐specific fractions from this analysis to corresponding super‐regions, and used global fractions for sub‐Saharan Africa.

### 2.5 HIV/AIDS misclassification correction

To correct for HIV deaths that were incorrectly assigned to other underlying causes (Appendix S1), we implemented the following steps: (1) examine the age patterns of underlying causes in location and years with and without HIV epidemics and isolate the causes with age pattern shifts during the epidemic years; (2) compute the expected deaths by location, year and sex for each underlying cause with an age‐pattern shift; (3) attribute expected deaths to the corresponding underlying cause; and (4) compute the difference between observed and expected deaths and reallocate the difference to HIV/AIDS [17].

To identify the age pattern in years without HIV epidemics, we generated a global standard relative mortality age pattern based on vital registration data from countries where HIV prevalence was less than 1% using the following equation:

$$RR_{asc}=\frac{R_{asc}}{\bar{x}\left(R_{65sc},R_{70sc},R_{75sc}\right)},$$

where $RR_{asc}$ is the relative death rate for age group *a*, sex *s* and cause *c*; $R_{asc}$ is the death rate for that age‐sex‐cause group; and $\bar{x}\left(R_{65sc},R_{70sc},R_{75sc}\right)$ is the average mortality rates in the 65–69, 70–74 and 75–79 age groups for that sex and cause.

Expected deaths for an identified underlying cause were computed using the following:

$$ED_{lyasc}=\bar{x}\left(R_{ly65sc},R_{ly70sc},R_{ly75sc}\right)\times p_{lasc}\times RR_{asc},$$

where $ED_{lyasc}$ are expected deaths for location *l*, year *y*, age group *a*, sex *s* and cause *c*; $\bar{x}\left(R_{ly65sc},R_{ly70sc},R_{ly75sc}\right)$ is the observed average cause‐specific mortality rates for the 65–69, 70–74 and 75–79 age groups for that location, year, sex and cause; $p_{lasc}$ is the population for that location, year, age, sex and cause; and $RR_{asc}$ is the global standard relative mortality rate determined during the preceding step.

## 3 RESULTS

### 3.1 Overall data description

Data on HIV deaths from 132 countries were subject to garbage code redistribution (Appendix S1). During redistribution, garbage codes that contributed deaths to HIV included data from all age groups, and 29 years by sex, yielding 646,528 unique records. During the period from 1990 to 2018, 1,848,761 deaths were reported as caused by HIV. After redistribution, this number increased to 4,165,015. Countries from seven super‐regions had redistributed deaths; the high‐income super‐region (*n* = 35) had the most countries with redistributed data, while South Asia had the least (*n* = 1). Results adjusted for vital registration completeness are available in Appendix S1. Countries with good quality vital registration systems were not largely affected by accounting for completeness.

HIV misclassification correction was more restricted than garbage code redistribution. The causes subject to HIV misclassification are shown in Appendix S1. These misclassified HIV deaths were located in 44 countries, primarily in central Europe, eastern Europe and central Asia (*n* = 16) and Latin America and the Caribbean (*n* = 16). Misclassification affected age groups up to age 65 and both sexes, for a total of 88,104 unique records. A total of 1,944,291 deaths were added through misclassification correction between 1990 and 2018. Data from children under 6 months of age were not subject to HIV correction.

The proportion of HIV deaths derived from redistribution declined over time, from 0.4 in 1990 to 0.1 in 2018 (Table 2). The proportion of deaths derived from HIV misclassification peaked at 0.4 in 2006 and gradually declined afterwards. Recent years had fewer countries with data, which tended to be higher quality, leading to lower proportions contributed by both redistribution and HIV correction in 2017 and 2018.

**Table 2:** Number and proportion of deaths corrected for garbage coding and HIV‐misclassification in 132 countries

Year	Raw deaths	Redistributed deaths	HIV corrected deaths	Proportion of redistributed deaths	Proportion of HIV corrected deaths	Proportion of raw deaths	Number of countries
1990	26,415	50,666	61,072	0.4	0.17	0.43	35
1991	29,623	59,044	69,882	0.42	0.16	0.42	35
1992	33,629	68,478	80,293	0.43	0.15	0.42	39
1993	37,838	76,333	83,804	0.46	0.09	0.45	37
1994	45,949	92,439	108,665	0.43	0.15	0.42	45
1995	48,477	125,382	150,426	0.51	0.17	0.32	55
1996	52,227	93,846	112,679	0.37	0.17	0.46	63
1997	44,468	110,185	147,878	0.44	0.25	0.3	72
1998	49,196	119,954	168,048	0.42	0.29	0.29	79
1999	60,269	143,781	203,953	0.41	0.3	0.3	86
2000	67,313	160,084	234,778	0.4	0.32	0.29	98
2001	56,447	148,246	223,955	0.41	0.34	0.25	99
2002	74,755	188,262	292,975	0.39	0.36	0.26	98
2003	78,695	206,751	323,758	0.4	0.36	0.24	98
2004	74,371	209,946	340,262	0.4	0.38	0.22	102
2005	71,251	207,498	339,453	0.4	0.39	0.21	102
2006	70,027	206,480	342,537	0.4	0.4	0.2	100
2007	82,399	219,530	349,428	0.39	0.37	0.24	102
2008	71,371	198,366	324,418	0.39	0.39	0.22	101
2009	74,869	195,959	309,312	0.39	0.37	0.24	105
2010	75,620	185,702	289,385	0.38	0.36	0.26	106
2011	75,556	171,214	261,594	0.37	0.35	0.29	104
2012	78,793	164,353	244,419	0.35	0.33	0.32	108
2013	92,316	167,916	241,086	0.31	0.3	0.38	107
2014	87,780	157,904	225,472	0.31	0.3	0.39	100
2015	90,457	160,631	223,352	0.31	0.28	0.4	92
2016	91,882	151,636	210,102	0.28	0.28	0.44	85
2017	65,067	77,675	95,227	0.13	0.18	0.68	69
2018	41,701	46,754	51,093	0.1	0.08	0.82	23

**Figure 1:**
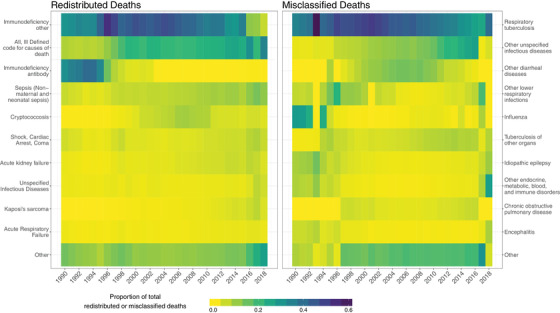
The distribution of the top ten contributors to redistributed and misclassified HIV deaths over time for all ages and sexes combined in included countries.

The greatest contributors to garbage code redistribution were “immunodeficiency antibody” and “immunodeficiency other,” whereas the largest contributor to misclassified HIV deaths was “Respiratory tuberculosis” over the most years (Figure 1).

### 3.2 Sources of misclassification and redistribution

**Figure 2:**
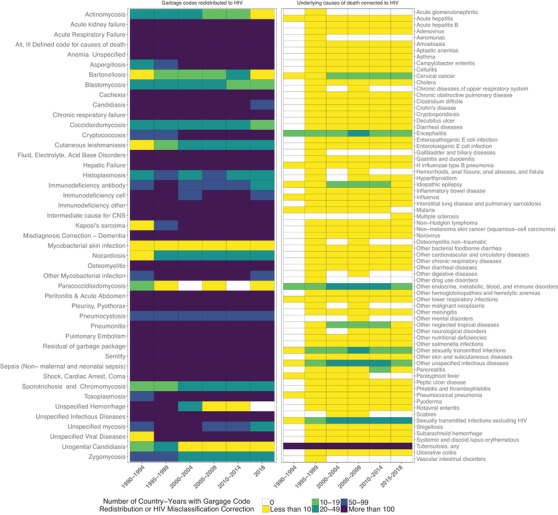
Variation in garbage code redistribution and HIV misclassification correction over time.

The number of countries with deaths from misclassification correction and redistribution, and contributing packages and causes, varied over time (Figure 2). The “mycobacterial skin infection” garbage code package contributed deaths in less than 10 country‐years in any given 5‐year period, while the “urogenital candidiasis” package contributed deaths to 15 country‐years between 1990 and 1994, but only one country‐year in 2010–2014. In contrast, only 10 causes contributed misclassified deaths in more than 10 country‐years. Finally, “tuberculosis, any” representing any form of tuberculosis, contributed deaths in over 100 country‐years in all 5‐year periods.

### 3.3 Patterns in redistribution and HIV misclassification correction with illustrative examples

**Figure 3:**
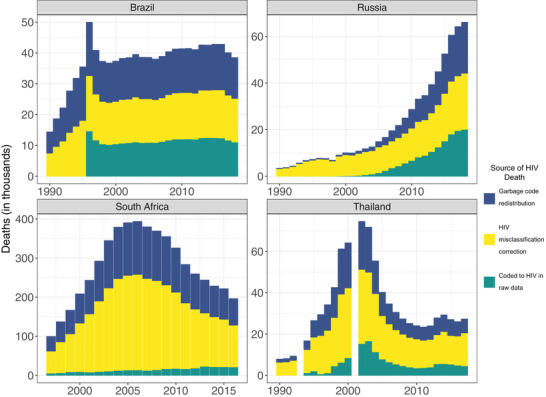
Raw data coded to HIV, garbage code redistributed and HIV misclassification corrected deaths in Brazil, Russia, South Africa and Thailand.

Fifty‐seven countries had at least 1 year between 1990 and 2018 when redistributed deaths contributed 100% of final deaths. The “immunodeficiency, other” ICD garbage code package (Table 1) contributed the largest number of redistributed deaths over the most years. This package contributed an average of 22,815 deaths per year across all included countries, which was 37% of all redistributed deaths. South Africa had the greatest number of deaths from redistribution in all years where it had data available (1997–2016), with an average of 36,985 deaths from redistribution per year (Figure 3). Mirroring the trend across all included countries, these deaths were mostly from the “immunodeficiency other” package. Prior to 1996, Brazil had the greatest number of deaths from redistribution, with an average of 17,529 deaths per year, mostly from the “immunodeficiency antibody” package. Less data from different countries are available for recent years; potentially as a result, Brazil and Russia had the most redistributed deaths in 2017 and 2018 (3061 in 2017 and 3405 in 2018 for Russia, and 3305 in 2017 and 3408 in 2018 for Brazil). These deaths were mostly from the “all, ill‐defined” ICD garbage code package.

HIV misclassification correction contributed between 8.5% and 39.7% of total deaths across all included countries, depending on the year, between 1990 and 2018. The deaths were mainly reassigned from “respiratory tuberculosis,” which contributed between 25% and 62% of all corrected deaths. The impact of HIV misclassification correction was more evident in some countries. For example, in Russia, more than 80% of final deaths were from HIV misclassification until 2004. This adjustment contributed an average of 6530 deaths per year to Russia's total death during this time period, which were primarily from “respiratory tuberculosis.” Misclassification correction was also common in Thailand, where it contributed between 34.7% and 79.3% of final deaths. This country had the most deaths from misclassification correction in most years prior to 1997; the adjustment contributed an average of 7087 deaths per year during this time. Similar to redistribution, South Africa then had the most deaths from misclassification correction between 1997 and 2016, with average of 70,294 deaths per year from misclassification (Figure 3). Also mirroring trends overall, these deaths were mostly from “respiratory tuberculosis.”

### 3.4 Effect of redistribution and HIV correction on age and sex distributions in HIV deaths

**Figure 4:**
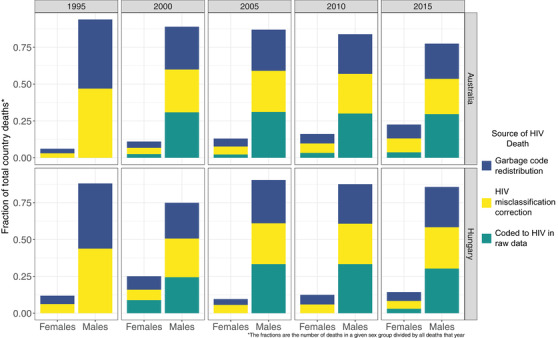
Raw data coded to HIV, garbage code redistributed and HIV misclassification corrected by sex in Australia and Hungary for 1995–2015.

Redistribution and HIV misclassification correction altered the sex and age distributions of HIV/AIDS deaths significantly in some countries. The effect of redistribution and misclassification correction on changing deaths between sexes is seen clearly in Australia and Hungary (Figure 4). In raw data from Australia, females had between 0 and 15 deaths between 1990 and 2017 compared to 0 to 463 deaths for males during the same period. After redistribution and misclassification correction, the proportion of deaths in females relative to males increased in some years. For example, in 2010, 64 out of 71 raw deaths were in males (90%) and 7 out of 71 raw deaths were in females (10%); after redistribution and misclassification correction, 79 out of 98 deaths were in males (81%) and the remaining were in females (19%). Hungary displayed this pattern as well. In 2010, 10 out of 10 raw deaths were in males (100%) and none were in females (0%); after redistribution and misclassification correction, 56 out of 67 deaths were in males (84%) and the remaining were in females (16%) (Figure 4).

**Figure 5:**
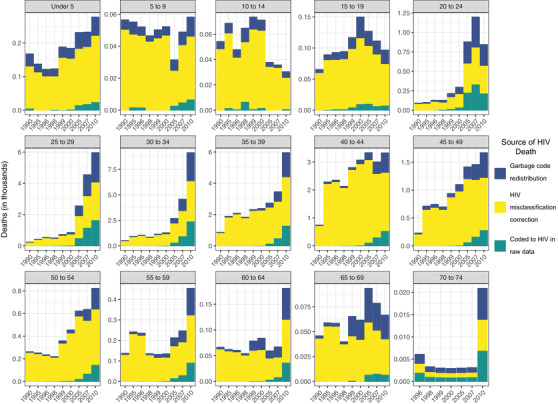
Raw data coded to HIV, garbage code redistributed and HIV misclassification corrected deaths by age in Russia for years 1990–2010.

Changes in age distribution were less pronounced and less common than changes in sex distribution. Reported versus adjusted deaths due to HIV in Russia illustrate the change in age distribution. Garbage code redistribution and HIV mis‐classification correction together shifted peak deaths in 1995 to older age groups, peaking only in 40‐ to 44‐year olds instead of having a second, smaller peak in 15‐ to 24‐year olds. The shift to older ages was a pattern in Russia; these adjusted data were more consistent with the reported data in later years after 2009 (Figure 5).

### 3.5 Effect of redistribution and HIV correction on time distributions in HIV deaths

**Figure 6:**
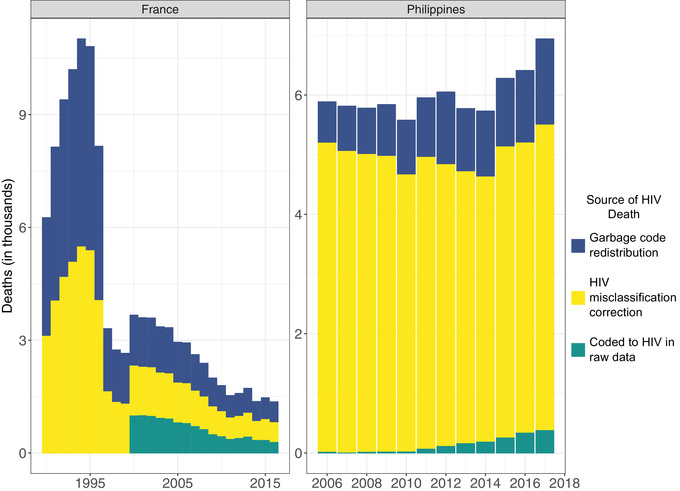
Raw data coded to HIV, garbage code redistributed and HIV misclassification corrected deaths over time in France and the Philippines.

More than sex and age, the time distributions were significantly altered by redistribution and/or misclassification, mirroring the distribution of underlying garbage codes and causes that contributed deaths. In several high‐income countries, the peak in HIV/AIDS deaths was shifted earlier from the mid‐1990s. This is seen in France's pre‐ and post‐adjustment death distributions, driven entirely by redistribution (Figure 6). Counter to expected trends, zero deaths were reported prior to 2000. Redistribution increased the mortality rate in the 1990s, primarily redistributed from the “immunodeficiency” garbage codes. In addition to the skewness of the death distribution, redistribution and misclassification also affected the shape of the mortality curve. This was evident in the Philippines, where the distribution of deaths was flattened over a longer period (Figure 6). The reported deaths suggested a sharp increase in HIV deaths between 2010 and 2017. The redistribution adjustment, driven by “non‐maternal and neonatal sepsis” and “shock, cardiac arrest and coma,” flattened the slope considerably. Misclassified deaths further flattened this distribution (Figure 6).

## 4 DISCUSSION

In this study, we have demonstrated the algorithms used to correct for garbage coding and HIV misclassification. These methods can be applied to country‐specific vital registration data to enhance the comparability of data within a country, between countries and over time. Our results showed that taking into account garbage coding and potential HIV misclassification could substantially enhance our understanding of the actual level and trend of country‐specific HIV‐related mortality.

Our results showed that the proportion of HIV deaths derived from redistribution of garbage codes have declined over time, indicating improvements in the cause of death certification and coding. Yet, there is considerable variation in the fraction of deaths assigned to garbage codes across countries globally. With the recent investments and initiatives, more training opportunities have become available in low‐ and middle‐income countries. As a part of the Bloomberg Philanthropies Data for Health Initiative Project [18], an evaluation of a strategy called “training of trainers” showed a decrease in incorrect completion of medical certificates of 28% in Sri Lanka and 40% in the Philippines [19]. The same study evaluating a different training strategy in Peru found a decrease in incorrect medical certificates of 43% after training of physicians on an online certification system and how to complete the certificate of cause of death [19].

Misclassification of HIV deaths as other underlying causes of death is common especially in non‐high‐income countries. Consistent with our results, studies have shown that HIV‐AIDS was commonly misclassified as tuberculosis and other infectious diseases [20, 21]. A study conducted in Thailand estimated that about two‐thirds of HIV deaths in the death registry during 1996–2009 did not record HIV as the underlying cause of death [20]. This finding is in line with our results, which showed that during the same period, garbage code redistribution and HIV misclassification correction together contributed between 49.9% and 96.2% of HIV deaths in Thailand. According to studies conducted in South Africa in 2000 and 2003–2004, HIV/AIDS was not recorded as the underlying cause of death for 61–73% of HIV/AIDS deaths [22, 23]. Bradshaw and colleagues estimated that 93% of HIV deaths were incorrectly assigned to other causes during the period from 1997 to 2010 in South Africa [24]. Consistently, our results showed that between 89.4% (in 1997) and 94.6% (in 2003) of HIV‐related deaths in South Africa were incorrectly assigned to garbage codes or other causes during the same period.

This study has some limitations. The quality of vital registration data varies across countries but we did not weight the data by data quality when calculating the global relative death rates. Additionally, multiple causes of death data were available only for a subset of countries. Due to the limited availability of data, we used the global redistribution proportions for sub‐Saharan Africa. Availability of additional multiple causes of death data will help to produce more accurate location‐specific redistribution proportions. In this study, we followed the ICD rule and considered certain infectious diseases associated with death, such as tuberculosis, among HIV‐positive people as a direct consequence of HIV/AIDS. It is possible that tuberculosis is not always a direct consequence of HIV/AIDS; for example, an HIV‐negative tuberculosis patient can be newly infected with HIV. As such data become available, they could be considered in future revisions of our methods. The cause of death data corrected for garbage coding and HIV‐misclassification (using the algorithms described in this paper) are used as inputs to the GBD modelling exercises, which produce results separately for overall HIV deaths and HIV‐tuberculosis deaths to facilitate an understanding of the mortality burden due to HIV and tuberculosis comorbidity.

## 5 CONCLUSIONS

Monitoring and tracking countries’ progress towards the global target of reducing HIV/AIDS‐related deaths by 90% between 2010 and 2030 [12, 13] requires reliable and valid HIV cause of death data. Whereas strengthening vital registration systems to accurately measure HIV‐related mortality is a principal public health goal for every country, until such a goal is achieved, statistical methods, such as those presented in this paper, will be required to enhance the utility of available cause‐of‐death data.

## FUNDING

This research was supported by funding from the Bill & Melinda Gates Foundation.

## COMPETING INTERESTS

The authors declare that they have no competing interests.

## AUTHORS' CONTRIBUTIONS

MN and CJLM provided overall guidance and helped devise the project and main conceptual ideas. HHK, DJ, MC, MW and NB prepared the first draft of the manuscript. MC, ID, FS, CH, SB and BG designed the underlying models and the computational framework, analysed the data and carried out experiments during the modelling process. HHK, DJ, MC, MW and NB contributed to the analysis of the results. EW and AN managed all technical aspects of the project pipeline and manuscript analyses. All authors provided critical feedback and helped shape the research, analysis and manuscript.

## Supporting information

**Appendix S1**: Overall data description ‐ Misclassification correction
