# Supplementary material for: Accounting for misclassified and unknown cause of death data in vital registration systems for estimating trends in HIV mortality
Source: J Int AIDS Soc. 2021 Sep 21;24(Suppl 5):e25791. doi: 10.1002/jia2.25791 (PMC8454675; doi:10.1002/jia2.25791)
Supplement: Supplementary file 1 — Appendix S1: Overall data description ‐ Misclassification correction [file JIA2-24-e25791-s001.docx]

Table of Contents

[Appendix Table 1. Raw, redistributed, and HIV-corrected deaths averaged by year 2](#_Toc76741708)

[Appendix Table 2. Raw, redistributed, and HIV-corrected deaths corrected for vital registration completeness, averaged by year 8](#_Toc76741709)

[Appendix Table 3. ICD9 Garbage Package ICD Codes 15](#_Toc76741710)

[Appendix Table 4. ICD10 Garbage Package ICD Codes 17](#_Toc76741711)

[Appendix Table 5. Misclassified HIV deaths, ICD 9 codes 19](#_Toc76741712)

[Appendix Table 6. Misclassified HIV deaths, ICD10 codes 22](#_Toc76741713)

[Appendix Table 7. Covariates used in multiple causes of death analysis 25](#_Toc76741714)

[Appendix Table 8. Redistribution Proportions for Pulmonary Embolism, All Ages Both Sexes, Year 2015, Top 20 Underlying Causes 25](#_Toc76741715)

[Appendix Figure 1. GBD Regions and Super Regions 28](#_Toc76741716)

[Appendix Figure 2. Overall causes-of-death data quality by country 29](#_Toc76741717)

[Appendix Figure 3. Variation in misclassification over time by super region. 30](#_Toc76741718)

[Estimating redistribution uncertainty 30](#_Toc76741719)

| Appendix Table 1. Raw, redistributed, and HIV-corrected deaths averaged by year |
| --- |

| **Country** | **Period** | **Raw Deaths** | **Redistributed Deaths** | **HIV Corrected Deaths** |
| --- | --- | --- | --- | --- |
| Armenia | 2000-2009 | 6 | 7 | 7 |
| Armenia | 2010-2018 | 26 | 29 | 29 |
| Azerbaijan | 2000-2009 | 0 | 20 | 41 |
| Georgia | 1990-1999 | 0 | 2 | 2 |
| Georgia | 2000-2009 | 1 | 2 | 2 |
| Georgia | 2010-2018 | 35 | 59 | 59 |
| Kazakhstan | 2010-2018 | 212 | 252 | 296 |
| Kyrgyzstan | 2000-2009 | 16 | 27 | 119 |
| Kyrgyzstan | 2010-2018 | 103 | 121 | 178 |
| Mongolia | 2010-2018 | 4 | 6 | 6 |
| Tajikistan | 2010-2018 | 0 | 1 | 65 |
| Uzbekistan | 2000-2009 | 73 | 102 | 335 |
| Uzbekistan | 2010-2018 | 290 | 336 | 482 |
| Albania | 1990-1999 | 0 | 3 | 3 |
| Albania | 2000-2009 | 0 | 1 | 1 |
| Albania | 2010-2018 | 0 | 0 | 0 |
| Bosnia and Herzegovina | 2010-2018 | 1 | 3 | 3 |
| Bulgaria | 1990-1999 | 0 | 3 | 57 |
| Bulgaria | 2000-2009 | 1 | 8 | 81 |
| Bulgaria | 2010-2018 | 12 | 22 | 66 |
| Croatia | 1990-1999 | 4 | 9 | 14 |
| Croatia | 2000-2009 | 5 | 7 | 10 |
| Croatia | 2010-2018 | 8 | 9 | 11 |
| Czech Republic | 1990-1999 | 2 | 7 | 7 |
| Czech Republic | 2000-2009 | 3 | 9 | 9 |
| Czech Republic | 2010-2018 | 11 | 21 | 21 |
| Hungary | 1990-1999 | 8 | 31 | 200 |
| Hungary | 2000-2009 | 8 | 18 | 102 |
| Hungary | 2010-2018 | 10 | 19 | 49 |
| Montenegro | 2000-2009 | 1 | 2 | 2 |
| North Macedonia | 1990-1999 | 0 | 0 | 0 |
| North Macedonia | 2000-2009 | 1 | 2 | 2 |
| North Macedonia | 2010-2018 | 0 | 2 | 2 |
| Poland | 1990-1999 | 16 | 37 | 37 |
| Poland | 2000-2009 | 117 | 161 | 161 |
| Poland | 2010-2018 | 118 | 158 | 158 |
| Romania | 1990-1999 | 484 | 516 | 519 |
| Romania | 2000-2009 | 274 | 291 | 292 |
| Romania | 2010-2018 | 183 | 216 | 216 |
| Serbia | 1990-1999 | 48 | 57 | 57 |
| Serbia | 2000-2009 | 29 | 37 | 37 |
| Serbia | 2010-2018 | 23 | 29 | 29 |
| Slovakia | 1990-1999 | 1 | 4 | 4 |
| Slovakia | 2000-2009 | 2 | 4 | 4 |
| Slovakia | 2010-2018 | 2 | 4 | 4 |
| Slovenia | 1990-1999 | 2 | 5 | 5 |
| Slovenia | 2000-2009 | 3 | 4 | 4 |
| Slovenia | 2010-2018 | 2 | 3 | 3 |
| Belarus | 2010-2018 | 266 | 283 | 343 |
| Estonia | 1990-1999 | 1 | 2 | 2 |
| Estonia | 2000-2009 | 26 | 28 | 28 |
| Estonia | 2010-2018 | 48 | 51 | 51 |
| Latvia | 1990-1999 | 1 | 5 | 92 |
| Latvia | 2000-2009 | 27 | 35 | 98 |
| Latvia | 2010-2018 | 86 | 93 | 146 |
| Lithuania | 1990-1999 | 1 | 4 | 54 |
| Lithuania | 2000-2009 | 8 | 12 | 80 |
| Lithuania | 2010-2018 | 24 | 30 | 81 |
| Moldova | 1990-1999 | 4 | 10 | 139 |
| Moldova | 2000-2009 | 38 | 45 | 191 |
| Moldova | 2010-2018 | 83 | 90 | 165 |
| Russia | 1990-1999 | 34 | 271 | 6029 |
| Russia | 2000-2009 | 2016 | 2739 | 10732 |
| Russia | 2010-2018 | 13359 | 14925 | 19054 |
| Ukraine | 2010-2018 | 2989 | 3186 | 4991 |
| Australia | 1990-1999 | 76 | 417 | 417 |
| Australia | 2000-2009 | 101 | 147 | 147 |
| Australia | 2010-2018 | 64 | 93 | 93 |
| New Zealand | 1990-1999 | 42 | 46 | 46 |
| New Zealand | 2000-2009 | 13 | 14 | 14 |
| New Zealand | 2010-2018 | 11 | 13 | 13 |
| Brunei | 2010-2018 | 2 | 4 | 4 |
| Japan | 1990-1999 | 36 | 141 | 141 |
| Japan | 2000-2009 | 63 | 211 | 211 |
| Japan | 2010-2018 | 54 | 228 | 228 |
| South Korea | 1990-1999 | 20 | 34 | 34 |
| South Korea | 2000-2009 | 71 | 88 | 88 |
| South Korea | 2010-2018 | 112 | 142 | 142 |
| Singapore | 1990-1999 | 0 | 30 | 30 |
| Singapore | 2000-2009 | 0 | 45 | 45 |
| Singapore | 2010-2018 | 29 | 37 | 37 |
| Canada | 1990-1999 | 0 | 943 | 943 |
| Canada | 2000-2009 | 429 | 483 | 483 |
| Canada | 2010-2018 | 233 | 271 | 271 |
| Greenland | 1990-1999 | 4 | 4 | 4 |
| Greenland | 2000-2009 | 3 | 3 | 3 |
| Greenland | 2010-2018 | 1 | 1 | 1 |
| USA | 1990-1999 | 28666 | 31783 | 31783 |
| USA | 2000-2009 | 12510 | 14077 | 14077 |
| USA | 2010-2018 | 6908 | 8010 | 8010 |
| Argentina | 1990-1999 | 1657 | 2000 | 2000 |
| Argentina | 2000-2009 | 1440 | 1782 | 1782 |
| Argentina | 2010-2018 | 1424 | 1789 | 1789 |
| Chile | 1990-1999 | 127 | 299 | 303 |
| Chile | 2000-2009 | 429 | 460 | 463 |
| Chile | 2010-2018 | 496 | 540 | 543 |
| Uruguay | 1990-1999 | 107 | 127 | 127 |
| Uruguay | 2000-2009 | 156 | 184 | 184 |
| Uruguay | 2010-2018 | 174 | 207 | 207 |
| Austria | 1990-1999 | 0 | 112 | 112 |
| Austria | 2000-2009 | 42 | 55 | 55 |
| Austria | 2010-2018 | 40 | 46 | 46 |
| Belgium | 1990-1999 | 14 | 142 | 142 |
| Belgium | 2000-2009 | 56 | 91 | 91 |
| Belgium | 2010-2018 | 42 | 83 | 83 |
| Cyprus | 2000-2009 | 2 | 3 | 3 |
| Cyprus | 2010-2018 | 2 | 4 | 4 |
| Denmark | 1990-1999 | 133 | 151 | 151 |
| Denmark | 2000-2009 | 33 | 43 | 43 |
| Denmark | 2010-2018 | 22 | 38 | 38 |
| Finland | 1990-1999 | 12 | 17 | 17 |
| Finland | 2000-2009 | 8 | 11 | 11 |
| Finland | 2010-2018 | 5 | 9 | 9 |
| France | 1990-1999 | 0 | 3634 | 3634 |
| France | 2000-2009 | 842 | 1100 | 1100 |
| France | 2010-2018 | 390 | 578 | 578 |
| Germany | 1990-1999 | 237 | 1325 | 1325 |
| Germany | 2000-2009 | 498 | 633 | 633 |
| Germany | 2010-2018 | 393 | 578 | 578 |
| Greece | 1990-1999 | 0 | 53 | 53 |
| Greece | 2000-2009 | 0 | 18 | 18 |
| Greece | 2010-2018 | 45 | 58 | 58 |
| Iceland | 1990-1999 | 0 | 3 | 3 |
| Iceland | 2000-2009 | 1 | 1 | 1 |
| Iceland | 2010-2018 | 0 | 1 | 1 |
| Ireland | 1990-1999 | 0 | 31 | 31 |
| Ireland | 2000-2009 | 5 | 14 | 14 |
| Ireland | 2010-2018 | 11 | 14 | 14 |
| Israel | 1990-1999 | 9 | 43 | 43 |
| Israel | 2000-2009 | 26 | 38 | 38 |
| Israel | 2010-2018 | 29 | 46 | 46 |
| Italy | 1990-1999 | 0 | 3074 | 3074 |
| Italy | 2000-2009 | 724 | 1116 | 1116 |
| Italy | 2010-2018 | 711 | 854 | 854 |
| Luxembourg | 1990-1999 | 1 | 7 | 7 |
| Luxembourg | 2000-2009 | 3 | 4 | 4 |
| Luxembourg | 2010-2018 | 4 | 4 | 4 |
| Malta | 1990-1999 | 2 | 4 | 4 |
| Malta | 2000-2009 | 1 | 2 | 2 |
| Malta | 2010-2018 | 3 | 3 | 3 |
| Monaco | 2010-2018 | 2 | 2 | 2 |
| Netherlands | 1990-1999 | 78 | 356 | 356 |
| Netherlands | 2000-2009 | 84 | 116 | 116 |
| Netherlands | 2010-2018 | 40 | 72 | 72 |
| Norway | 1990-1999 | 11 | 46 | 46 |
| Norway | 2000-2009 | 16 | 20 | 20 |
| Norway | 2010-2018 | 10 | 17 | 17 |
| Portugal | 1990-1999 | 0 | 854 | 854 |
| Portugal | 2000-2009 | 631 | 973 | 973 |
| Portugal | 2010-2018 | 450 | 532 | 532 |
| San Marino | 1990-1999 | 0 | 0 | 0 |
| San Marino | 2000-2009 | 0 | 0 | 0 |
| Spain | 1990-1999 | 180 | 3737 | 3737 |
| Spain | 2000-2009 | 1450 | 1612 | 1612 |
| Spain | 2010-2018 | 734 | 858 | 858 |
| Sweden | 1990-1999 | 10 | 90 | 90 |
| Sweden | 2000-2009 | 26 | 34 | 34 |
| Sweden | 2010-2018 | 14 | 27 | 27 |
| Switzerland | 1990-1999 | 333 | 367 | 367 |
| Switzerland | 2000-2009 | 85 | 100 | 100 |
| Switzerland | 2010-2018 | 37 | 49 | 49 |
| UK | 1990-1999 | 237 | 574 | 574 |
| UK | 2000-2009 | 232 | 324 | 324 |
| UK | 2010-2018 | 193 | 297 | 297 |
| Bolivia | 2000-2009 | 9 | 95 | 95 |
| Ecuador | 1990-1999 | 141 | 269 | 269 |
| Ecuador | 2000-2009 | 524 | 668 | 668 |
| Ecuador | 2010-2018 | 767 | 878 | 878 |
| Peru | 1990-1999 | 406 | 1199 | 1199 |
| Peru | 2000-2009 | 836 | 1208 | 1208 |
| Peru | 2010-2018 | 789 | 1080 | 1080 |
| Antigua and Barbuda | 1990-1999 | 7 | 9 | 9 |
| Antigua and Barbuda | 2000-2009 | 10 | 11 | 11 |
| Antigua and Barbuda | 2010-2018 | 7 | 10 | 10 |
| The Bahamas | 1990-1999 | 275 | 285 | 285 |
| The Bahamas | 2000-2009 | 177 | 187 | 187 |
| The Bahamas | 2010-2018 | 110 | 122 | 122 |
| Barbados | 2000-2009 | 33 | 40 | 40 |
| Barbados | 2010-2018 | 8 | 19 | 19 |
| Belize | 1990-1999 | 30 | 38 | 38 |
| Belize | 2000-2009 | 71 | 77 | 77 |
| Belize | 2010-2018 | 108 | 114 | 114 |
| Bermuda | 1990-1999 | 16 | 17 | 17 |
| Bermuda | 2000-2009 | 8 | 8 | 8 |
| Bermuda | 2010-2018 | 2 | 2 | 2 |
| Cuba | 1990-1999 | 0 | 118 | 118 |
| Cuba | 2000-2009 | 114 | 138 | 138 |
| Cuba | 2010-2018 | 355 | 378 | 378 |
| Dominica | 2000-2009 | 6 | 9 | 9 |
| Dominica | 2010-2018 | 3 | 6 | 6 |
| Dominican Republic | 1990-1999 | 902 | 1195 | 1195 |
| Dominican Republic | 2000-2009 | 804 | 920 | 920 |
| Dominican Republic | 2010-2018 | 537 | 630 | 630 |
| Grenada | 2000-2009 | 6 | 7 | 7 |
| Grenada | 2010-2018 | 5 | 8 | 8 |
| Guyana | 1990-1999 | 0 | 67 | 67 |
| Guyana | 2000-2009 | 404 | 423 | 423 |
| Guyana | 2010-2018 | 211 | 229 | 229 |
| Haiti | 1990-1999 | 1280 | 2585 | 2585 |
| Haiti | 2000-2009 | 378 | 613 | 613 |
| Jamaica | 2000-2009 | 464 | 642 | 670 |
| Jamaica | 2010-2018 | 535 | 564 | 579 |
| Puerto Rico | 1990-1999 | 1132 | 1256 | 1256 |
| Puerto Rico | 2000-2009 | 515 | 587 | 587 |
| Puerto Rico | 2010-2018 | 243 | 296 | 296 |
| Saint Kitts and Nevis | 1990-1999 | 4 | 8 | 8 |
| Saint Kitts and Nevis | 2000-2009 | 2 | 4 | 4 |
| Saint Kitts and Nevis | 2010-2018 | 3 | 4 | 4 |
| Saint Lucia | 1990-1999 | 8 | 10 | 10 |
| Saint Lucia | 2000-2009 | 9 | 11 | 11 |
| Saint Lucia | 2010-2018 | 4 | 6 | 7 |
| Saint Vincent and the Grenadines | 1990-1999 | 0 | 2 | 2 |
| Saint Vincent and the Grenadines | 2000-2009 | 30 | 33 | 33 |
| Saint Vincent and the Grenadines | 2010-2018 | 17 | 24 | 24 |
| Suriname | 1990-1999 | 51 | 70 | 70 |
| Suriname | 2000-2009 | 133 | 150 | 150 |
| Suriname | 2010-2018 | 101 | 114 | 114 |
| Trinidad and Tobago | 1990-1999 | 519 | 555 | 555 |
| Trinidad and Tobago | 2000-2009 | 362 | 385 | 385 |
| Trinidad and Tobago | 2010-2018 | 221 | 238 | 238 |
| Virgin Islands | 1990-1999 | 18 | 21 | 21 |
| Virgin Islands | 2000-2009 | 7 | 9 | 9 |
| Virgin Islands | 2010-2018 | 6 | 8 | 8 |
| Colombia | 1990-1999 | 462 | 700 | 1201 |
| Colombia | 2000-2009 | 2263 | 2482 | 2656 |
| Colombia | 2010-2018 | 2454 | 2685 | 2821 |
| Costa Rica | 1990-1999 | 143 | 154 | 167 |
| Costa Rica | 2000-2009 | 122 | 132 | 154 |
| Costa Rica | 2010-2018 | 147 | 161 | 186 |
| El Salvador | 1990-1999 | 319 | 413 | 413 |
| El Salvador | 2000-2009 | 464 | 562 | 562 |
| El Salvador | 2010-2018 | 286 | 386 | 386 |
| Guatemala | 2000-2009 | 339 | 675 | 735 |
| Guatemala | 2010-2018 | 428 | 525 | 587 |
| Honduras | 2000-2009 | 154 | 163 | 193 |
| Honduras | 2010-2018 | 149 | 159 | 184 |
| Mexico | 1990-1999 | 773 | 3435 | 3759 |
| Mexico | 2000-2009 | 4427 | 4814 | 5136 |
| Mexico | 2010-2018 | 4797 | 5251 | 5688 |
| Nicaragua | 1990-1999 | 21 | 29 | 29 |
| Nicaragua | 2000-2009 | 80 | 94 | 94 |
| Nicaragua | 2010-2018 | 219 | 238 | 238 |
| Panama | 1990-1999 | 442 | 493 | 493 |
| Panama | 2000-2009 | 461 | 507 | 507 |
| Panama | 2010-2018 | 511 | 574 | 574 |
| Venezuela | 1990-1999 | 1093 | 1183 | 1208 |
| Venezuela | 2000-2009 | 1436 | 1514 | 1548 |
| Venezuela | 2010-2018 | 2080 | 2163 | 2196 |
| Brazil | 1990-1999 | 4835 | 13338 | 14258 |
| Brazil | 2000-2009 | 11249 | 13704 | 14600 |
| Brazil | 2010-2018 | 12154 | 14350 | 15264 |
| Paraguay | 1990-1999 | 39 | 67 | 67 |
| Paraguay | 2000-2009 | 141 | 180 | 180 |
| Paraguay | 2010-2018 | 218 | 260 | 260 |
| Algeria | 2000-2009 | 14 | 48 | 48 |
| Bahrain | 1990-1999 | 11 | 13 | 13 |
| Bahrain | 2000-2009 | 10 | 13 | 13 |
| Bahrain | 2010-2018 | 8 | 12 | 12 |
| Egypt | 2000-2009 | 6 | 262 | 262 |
| Egypt | 2010-2018 | 11 | 388 | 388 |
| Iran | 2000-2009 | 118 | 155 | 155 |
| Iran | 2010-2018 | 258 | 417 | 417 |
| Iraq | 2000-2009 | 0 | 60 | 60 |
| Iraq | 2010-2018 | 5 | 71 | 71 |
| Jordan | 2000-2009 | 3 | 12 | 12 |
| Jordan | 2010-2018 | 1 | 11 | 11 |
| Kuwait | 1990-1999 | 0 | 2 | 2 |
| Kuwait | 2000-2009 | 2 | 3 | 3 |
| Kuwait | 2010-2018 | 2 | 3 | 3 |
| Lebanon | 2010-2018 | 11 | 25 | 25 |
| Morocco | 2000-2009 | 23 | 78 | 78 |
| Morocco | 2010-2018 | 40 | 91 | 91 |
| Oman | 2000-2009 | 16 | 18 | 19 |
| Oman | 2010-2018 | 7 | 22 | 30 |
| Palestine | 2000-2009 | 0 | 3 | 3 |
| Palestine | 2010-2018 | 0 | 6 | 6 |
| Qatar | 1990-1999 | 7 | 9 | 9 |
| Qatar | 2000-2009 | 2 | 4 | 4 |
| Qatar | 2010-2018 | 1 | 3 | 3 |
| Saudi Arabia | 1990-1999 | 61 | 85 | 85 |
| Saudi Arabia | 2000-2009 | 113 | 170 | 170 |
| Saudi Arabia | 2010-2018 | 139 | 228 | 228 |
| Syria | 2010-2018 | 0 | 75 | 75 |
| Tunisia | 2000-2009 | 14 | 25 | 25 |
| Tunisia | 2010-2018 | 7 | 25 | 25 |
| Turkey | 2000-2009 | 44 | 120 | 120 |
| Turkey | 2010-2018 | 69 | 172 | 172 |
| India | 2000-2009 | 15 | 167 | 167 |
| India | 2010-2018 | 58 | 160 | 160 |
| China | 1990-1999 | 0 | 13 | 13 |
| China | 2000-2009 | 495 | 632 | 653 |
| China | 2010-2018 | 4142 | 5122 | 5181 |
| Taiwan (province of China) | 1990-1999 | 41 | 56 | 56 |
| Taiwan (province of China) | 2000-2009 | 94 | 119 | 119 |
| Taiwan (province of China) | 2010-2018 | 149 | 194 | 194 |
| American Samoa | 1990-1999 | 0 | 1 | 1 |
| American Samoa | 2000-2009 | 0 | 1 | 1 |
| American Samoa | 2010-2018 | 0 | 1 | 1 |
| Fiji | 2000-2009 | 3 | 53 | 53 |
| Fiji | 2010-2018 | 2 | 27 | 27 |
| Guam | 1990-1999 | 4 | 5 | 5 |
| Guam | 2000-2009 | 3 | 5 | 5 |
| Guam | 2010-2018 | 2 | 6 | 6 |
| Kiribati | 1990-1999 | 0 | 11 | 11 |
| Kiribati | 2000-2009 | 0 | 6 | 6 |
| Northern Mariana Islands | 1990-1999 | 0 | 1 | 1 |
| Northern Mariana Islands | 2000-2009 | 0 | 2 | 2 |
| Northern Mariana Islands | 2010-2018 | 0 | 1 | 1 |
| Palau | 2010-2018 | 0 | 3 | 3 |
| Solomon Islands | 2010-2018 | 0 | 3 | 3 |
| Malaysia | 1990-1999 | 0 | 653 | 1076 |
| Malaysia | 2000-2009 | 387 | 836 | 1613 |
| Malaysia | 2010-2018 | 564 | 911 | 1277 |
| Maldives | 2000-2009 | 0 | 2 | 2 |
| Maldives | 2010-2018 | 0 | 4 | 4 |
| Mauritius | 1990-1999 | 0 | 3 | 3 |
| Mauritius | 2000-2009 | 19 | 29 | 29 |
| Mauritius | 2010-2018 | 97 | 106 | 106 |
| Philippines | 2000-2009 | 26 | 757 | 5047 |
| Philippines | 2010-2018 | 202 | 1122 | 4766 |
| Sri Lanka | 2000-2009 | 1 | 124 | 124 |
| Sri Lanka | 2010-2018 | 2 | 118 | 118 |
| Thailand | 1990-1999 | 1838 | 8564 | 16713 |
| Thailand | 2000-2009 | 9045 | 14130 | 24402 |
| Thailand | 2010-2018 | 4729 | 7081 | 14367 |
| South Africa | 1990-1999 | 7790 | 51334 | 80430 |
| South Africa | 2000-2009 | 13174 | 119253 | 211059 |
| South Africa | 2010-2018 | 20855 | 87877 | 145553 |
| Zimbabwe | 1990-1999 | 0 | 20430 | 27295 |
| Zimbabwe | 2000-2009 | 13068 | 16505 | 18718 |
| Cape Verde | 2010-2018 | 70 | 89 | 89 |
| Ghana | 2000-2009 | 607 | 1018 | 1270 |

## Appendix Table 2. Raw, redistributed, and HIV-corrected deaths corrected for vital registration completeness, averaged by year

| **Country** | **Period** | **Raw Deaths** | **Redistributed Deaths** | **HIV Corrected Deaths** |
| --- | --- | --- | --- | --- |
| Armenia | 2000-2009 | 6 | 7 | 7 |
| Armenia | 2010-2018 | 27 | 30 | 30 |
| Azerbaijan | 2000-2009 | 0 | 27 | 56 |
| Georgia | 1990-1999 | 0 | 2 | 2 |
| Georgia | 2000-2009 | 1 | 3 | 3 |
| Georgia | 2010-2018 | 42 | 68 | 68 |
| Kazakhstan | 2010-2018 | 223 | 266 | 312 |
| Kyrgyzstan | 2000-2009 | 17 | 29 | 128 |
| Kyrgyzstan | 2010-2018 | 107 | 125 | 184 |
| Mongolia | 2010-2018 | 5 | 9 | 9 |
| Tajikistan | 2010-2018 | 0 | 1 | 96 |
| Uzbekistan | 2000-2009 | 111 | 155 | 512 |
| Uzbekistan | 2010-2018 | 401 | 464 | 669 |
| Albania | 1990-1999 | 0 | 3 | 3 |
| Albania | 2000-2009 | 0 | 1 | 1 |
| Albania | 2010-2018 | 0 | 0 | 0 |
| Bosnia and Herzegovina | 2010-2018 | 1 | 3 | 3 |
| Bulgaria | 1990-1999 | 0 | 4 | 63 |
| Bulgaria | 2000-2009 | 1 | 9 | 91 |
| Bulgaria | 2010-2018 | 13 | 24 | 74 |
| Croatia | 1990-1999 | 4 | 9 | 14 |
| Croatia | 2000-2009 | 5 | 7 | 10 |
| Croatia | 2010-2018 | 8 | 9 | 11 |
| Czechia | 1990-1999 | 2 | 7 | 7 |
| Czechia | 2000-2009 | 3 | 9 | 9 |
| Czechia | 2010-2018 | 11 | 21 | 21 |
| Hungary | 1990-1999 | 8 | 31 | 200 |
| Hungary | 2000-2009 | 8 | 18 | 102 |
| Hungary | 2010-2018 | 10 | 19 | 49 |
| Montenegro | 2000-2009 | 1 | 2 | 2 |
| North Macedonia | 1990-1999 | 0 | 0 | 0 |
| North Macedonia | 2000-2009 | 1 | 2 | 2 |
| North Macedonia | 2010-2018 | 0 | 2 | 2 |
| Poland | 1990-1999 | 16 | 37 | 37 |
| Poland | 2000-2009 | 117 | 161 | 161 |
| Poland | 2010-2018 | 118 | 158 | 158 |
| Romania | 1990-1999 | 484 | 516 | 519 |
| Romania | 2000-2009 | 274 | 291 | 292 |
| Romania | 2010-2018 | 183 | 216 | 216 |
| Serbia | 1990-1999 | 52 | 63 | 63 |
| Serbia | 2000-2009 | 32 | 41 | 41 |
| Serbia | 2010-2018 | 25 | 33 | 33 |
| Slovakia | 1990-1999 | 1 | 4 | 4 |
| Slovakia | 2000-2009 | 2 | 4 | 4 |
| Slovakia | 2010-2018 | 2 | 4 | 4 |
| Slovenia | 1990-1999 | 2 | 5 | 5 |
| Slovenia | 2000-2009 | 3 | 4 | 4 |
| Slovenia | 2010-2018 | 2 | 3 | 3 |
| Belarus | 2010-2018 | 279 | 297 | 361 |
| Estonia | 1990-1999 | 1 | 2 | 2 |
| Estonia | 2000-2009 | 26 | 28 | 28 |
| Estonia | 2010-2018 | 48 | 51 | 51 |
| Latvia | 1990-1999 | 1 | 5 | 92 |
| Latvia | 2000-2009 | 27 | 35 | 99 |
| Latvia | 2010-2018 | 86 | 93 | 146 |
| Lithuania | 1990-1999 | 1 | 4 | 54 |
| Lithuania | 2000-2009 | 8 | 12 | 81 |
| Lithuania | 2010-2018 | 24 | 30 | 81 |
| Republic of Moldova | 1990-1999 | 5 | 11 | 146 |
| Republic of Moldova | 2000-2009 | 42 | 50 | 208 |
| Republic of Moldova | 2010-2018 | 92 | 101 | 183 |
| Russian Federation | 1990-1999 | 31 | 257 | 5752 |
| Russian Federation | 2000-2009 | 1889 | 2579 | 10288 |
| Russian Federation | 2010-2018 | 12764 | 14283 | 18259 |
| Ukraine | 2010-2018 | 2434 | 2602 | 4006 |
| Australia | 1990-1999 | 76 | 417 | 417 |
| Australia | 2000-2009 | 101 | 147 | 147 |
| Australia | 2010-2018 | 64 | 93 | 93 |
| New Zealand | 1990-1999 | 43 | 47 | 47 |
| New Zealand | 2000-2009 | 13 | 15 | 15 |
| New Zealand | 2010-2018 | 11 | 13 | 13 |
| Brunei Darussalam | 2010-2018 | 3 | 5 | 5 |
| Japan | 1990-1999 | 36 | 141 | 141 |
| Japan | 2000-2009 | 63 | 212 | 212 |
| Japan | 2010-2018 | 54 | 228 | 228 |
| Republic of Korea | 1990-1999 | 20 | 34 | 34 |
| Republic of Korea | 2000-2009 | 71 | 88 | 88 |
| Republic of Korea | 2010-2018 | 112 | 142 | 142 |
| Singapore | 1990-1999 | 0 | 30 | 30 |
| Singapore | 2000-2009 | 0 | 45 | 45 |
| Singapore | 2010-2018 | 29 | 37 | 37 |
| Canada | 1990-1999 | 0 | 943 | 943 |
| Canada | 2000-2009 | 429 | 483 | 483 |
| Canada | 2010-2018 | 233 | 271 | 271 |
| Greenland | 1990-1999 | 4 | 4 | 4 |
| Greenland | 2000-2009 | 3 | 3 | 3 |
| Greenland | 2010-2018 | 1 | 1 | 1 |
| United States of America | 1990-1999 | 28667 | 31784 | 31784 |
| United States of America | 2000-2009 | 13903 | 15586 | 15586 |
| Argentina | 1990-1999 | 1657 | 2000 | 2000 |
| Argentina | 2000-2009 | 1440 | 1782 | 1782 |
| Argentina | 2010-2018 | 1424 | 1789 | 1789 |
| Chile | 1990-1999 | 127 | 300 | 304 |
| Chile | 2000-2009 | 432 | 464 | 467 |
| Chile | 2010-2018 | 497 | 541 | 544 |
| Uruguay | 1990-1999 | 107 | 127 | 127 |
| Uruguay | 2000-2009 | 156 | 185 | 185 |
| Uruguay | 2010-2018 | 174 | 207 | 207 |
| Austria | 1990-1999 | 0 | 112 | 112 |
| Austria | 2000-2009 | 42 | 55 | 55 |
| Austria | 2010-2018 | 40 | 46 | 46 |
| Belgium | 1990-1999 | 14 | 142 | 142 |
| Belgium | 2000-2009 | 56 | 91 | 91 |
| Belgium | 2010-2018 | 42 | 83 | 83 |
| Cyprus | 2000-2009 | 2 | 4 | 4 |
| Cyprus | 2010-2018 | 3 | 6 | 6 |
| Denmark | 1990-1999 | 133 | 151 | 151 |
| Denmark | 2000-2009 | 33 | 43 | 43 |
| Denmark | 2010-2018 | 22 | 38 | 38 |
| Finland | 1990-1999 | 12 | 17 | 17 |
| Finland | 2000-2009 | 8 | 11 | 11 |
| Finland | 2010-2018 | 5 | 9 | 9 |
| France | 1990-1999 | 0 | 3634 | 3634 |
| France | 2000-2009 | 842 | 1100 | 1100 |
| France | 2010-2018 | 390 | 578 | 578 |
| Germany | 1990-1999 | 237 | 1325 | 1325 |
| Germany | 2000-2009 | 498 | 633 | 633 |
| Germany | 2010-2018 | 393 | 578 | 578 |
| Greece | 1990-1999 | 0 | 53 | 53 |
| Greece | 2000-2009 | 0 | 18 | 18 |
| Greece | 2010-2018 | 45 | 58 | 58 |
| Iceland | 1990-1999 | 0 | 3 | 3 |
| Iceland | 2000-2009 | 1 | 1 | 1 |
| Iceland | 2010-2018 | 0 | 1 | 1 |
| Ireland | 1990-1999 | 0 | 31 | 31 |
| Ireland | 2000-2009 | 5 | 14 | 14 |
| Ireland | 2010-2018 | 11 | 14 | 14 |
| Israel | 1990-1999 | 9 | 43 | 43 |
| Israel | 2000-2009 | 26 | 38 | 38 |
| Israel | 2010-2018 | 29 | 46 | 46 |
| Italy | 1990-1999 | 0 | 3074 | 3074 |
| Italy | 2000-2009 | 725 | 1117 | 1117 |
| Italy | 2010-2018 | 711 | 855 | 855 |
| Luxembourg | 1990-1999 | 1 | 7 | 7 |
| Luxembourg | 2000-2009 | 3 | 4 | 4 |
| Luxembourg | 2010-2018 | 4 | 4 | 4 |
| Malta | 1990-1999 | 2 | 4 | 4 |
| Malta | 2000-2009 | 1 | 2 | 2 |
| Malta | 2010-2018 | 3 | 3 | 3 |
| Monaco | 2010-2018 | 2 | 2 | 2 |
| Netherlands | 1990-1999 | 78 | 356 | 356 |
| Netherlands | 2000-2009 | 84 | 116 | 116 |
| Netherlands | 2010-2018 | 40 | 72 | 72 |
| Norway | 1990-1999 | 11 | 46 | 46 |
| Norway | 2000-2009 | 16 | 21 | 21 |
| Norway | 2010-2018 | 10 | 17 | 17 |
| Portugal | 1990-1999 | 0 | 854 | 854 |
| Portugal | 2000-2009 | 634 | 977 | 977 |
| Portugal | 2010-2018 | 450 | 532 | 532 |
| San Marino | 1990-1999 | 0 | 0 | 0 |
| San Marino | 2000-2009 | 0 | 0 | 0 |
| Spain | 1990-1999 | 180 | 3737 | 3737 |
| Spain | 2000-2009 | 1450 | 1612 | 1612 |
| Spain | 2010-2018 | 734 | 858 | 858 |
| Sweden | 1990-1999 | 10 | 90 | 90 |
| Sweden | 2000-2009 | 26 | 34 | 34 |
| Sweden | 2010-2018 | 14 | 27 | 27 |
| Switzerland | 1990-1999 | 333 | 367 | 367 |
| Switzerland | 2000-2009 | 85 | 100 | 100 |
| Switzerland | 2010-2018 | 37 | 49 | 49 |
| United Kingdom | 1990-1999 | 236 | 498 | 498 |
| United Kingdom | 2000-2009 | 203 | 284 | 284 |
| United Kingdom | 2010-2018 | 176 | 266 | 266 |
| Bolivia (Plurinational State of) | 2000-2009 | 23 | 255 | 255 |
| Ecuador | 1990-1999 | 149 | 284 | 284 |
| Ecuador | 2000-2009 | 659 | 835 | 835 |
| Ecuador | 2010-2018 | 988 | 1132 | 1132 |
| Peru | 1990-1999 | 563 | 1664 | 1664 |
| Peru | 2000-2009 | 1357 | 1738 | 1738 |
| Peru | 2010-2018 | 1114 | 1522 | 1522 |
| Antigua and Barbuda | 1990-1999 | 7 | 9 | 9 |
| Antigua and Barbuda | 2000-2009 | 10 | 12 | 12 |
| Antigua and Barbuda | 2010-2018 | 8 | 11 | 11 |
| Bahamas | 1990-1999 | 281 | 291 | 291 |
| Bahamas | 2000-2009 | 178 | 188 | 188 |
| Bahamas | 2010-2018 | 113 | 125 | 125 |
| Barbados | 2000-2009 | 35 | 43 | 43 |
| Barbados | 2010-2018 | 9 | 20 | 20 |
| Belize | 1990-1999 | 31 | 39 | 39 |
| Belize | 2000-2009 | 71 | 77 | 77 |
| Belize | 2010-2018 | 108 | 114 | 114 |
| Bermuda | 1990-1999 | 16 | 17 | 17 |
| Bermuda | 2000-2009 | 8 | 8 | 8 |
| Bermuda | 2010-2018 | 2 | 2 | 2 |
| Cuba | 1990-1999 | 0 | 118 | 118 |
| Cuba | 2000-2009 | 114 | 138 | 138 |
| Cuba | 2010-2018 | 355 | 378 | 378 |
| Dominica | 2000-2009 | 7 | 11 | 11 |
| Dominica | 2010-2018 | 4 | 7 | 7 |
| Dominican Republic | 1990-1999 | 1411 | 1870 | 1870 |
| Dominican Republic | 2000-2009 | 1658 | 1892 | 1892 |
| Dominican Republic | 2010-2018 | 923 | 1082 | 1082 |
| Grenada | 2000-2009 | 6 | 7 | 7 |
| Grenada | 2010-2018 | 5 | 8 | 8 |
| Guyana | 1990-1999 | 0 | 91 | 91 |
| Guyana | 2000-2009 | 473 | 496 | 496 |
| Guyana | 2010-2018 | 237 | 257 | 257 |
| Jamaica | 2000-2009 | 471 | 651 | 680 |
| Jamaica | 2010-2018 | 549 | 580 | 595 |
| Puerto Rico | 1990-1999 | 1132 | 1256 | 1256 |
| Puerto Rico | 2000-2009 | 515 | 587 | 587 |
| Puerto Rico | 2010-2018 | 244 | 296 | 296 |
| Saint Kitts and Nevis | 1990-1999 | 4 | 8 | 8 |
| Saint Kitts and Nevis | 2000-2009 | 3 | 5 | 5 |
| Saint Kitts and Nevis | 2010-2018 | 4 | 4 | 4 |
| Saint Lucia | 1990-1999 | 8 | 10 | 10 |
| Saint Lucia | 2000-2009 | 9 | 11 | 11 |
| Saint Lucia | 2010-2018 | 4 | 7 | 7 |
| Saint Vincent and the Grenadines | 1990-1999 | 0 | 3 | 3 |
| Saint Vincent and the Grenadines | 2000-2009 | 30 | 34 | 34 |
| Saint Vincent and the Grenadines | 2010-2018 | 18 | 24 | 24 |
| Suriname | 1990-1999 | 62 | 86 | 86 |
| Suriname | 2000-2009 | 155 | 175 | 175 |
| Suriname | 2010-2018 | 116 | 130 | 130 |
| Trinidad and Tobago | 1990-1999 | 519 | 555 | 555 |
| Trinidad and Tobago | 2000-2009 | 362 | 385 | 385 |
| Trinidad and Tobago | 2010-2018 | 221 | 238 | 238 |
| United States Virgin Islands | 1990-1999 | 21 | 24 | 24 |
| United States Virgin Islands | 2000-2009 | 9 | 12 | 12 |
| United States Virgin Islands | 2010-2018 | 10 | 13 | 13 |
| Colombia | 1990-1999 | 472 | 724 | 1254 |
| Colombia | 2000-2009 | 2281 | 2502 | 2678 |
| Colombia | 2010-2018 | 2457 | 2687 | 2823 |
| Costa Rica | 1990-1999 | 143 | 154 | 167 |
| Costa Rica | 2000-2009 | 122 | 132 | 154 |
| Costa Rica | 2010-2018 | 147 | 161 | 186 |
| El Salvador | 1990-1999 | 354 | 457 | 457 |
| El Salvador | 2000-2009 | 492 | 596 | 596 |
| El Salvador | 2010-2018 | 291 | 392 | 392 |
| Guatemala | 2000-2009 | 362 | 720 | 784 |
| Guatemala | 2010-2018 | 456 | 558 | 623 |
| Mexico | 1990-1999 | 848 | 3811 | 4181 |
| Mexico | 2000-2009 | 4789 | 5209 | 5559 |
| Mexico | 2010-2018 | 4859 | 5319 | 5761 |
| Nicaragua | 1990-1999 | 26 | 36 | 36 |
| Nicaragua | 2000-2009 | 96 | 112 | 112 |
| Nicaragua | 2010-2018 | 242 | 263 | 263 |
| Panama | 1990-1999 | 442 | 493 | 493 |
| Panama | 2000-2009 | 461 | 507 | 507 |
| Panama | 2010-2018 | 511 | 574 | 574 |
| Venezuela (Bolivarian Republic of) | 1990-1999 | 1094 | 1184 | 1209 |
| Venezuela (Bolivarian Republic of) | 2000-2009 | 1459 | 1538 | 1573 |
| Venezuela (Bolivarian Republic of) | 2010-2018 | 2087 | 2170 | 2203 |
| Brazil | 1990-1999 | 5042 | 14192 | 15331 |
| Brazil | 2000-2009 | 11811 | 14522 | 15547 |
| Brazil | 2010-2018 | 12547 | 14834 | 15796 |
| Paraguay | 1990-1999 | 45 | 78 | 78 |
| Paraguay | 2000-2009 | 157 | 202 | 202 |
| Paraguay | 2010-2018 | 235 | 281 | 281 |
| Bahrain | 1990-1999 | 13 | 15 | 15 |
| Bahrain | 2000-2009 | 11 | 15 | 15 |
| Bahrain | 2010-2018 | 9 | 15 | 15 |
| Iran (Islamic Republic of) | 2000-2009 | 135 | 176 | 176 |
| Iran (Islamic Republic of) | 2010-2018 | 296 | 481 | 481 |
| Iraq | 2000-2009 | 0 | 87 | 87 |
| Iraq | 2010-2018 | 7 | 103 | 103 |
| Jordan | 2000-2009 | 3 | 14 | 14 |
| Jordan | 2010-2018 | 2 | 14 | 14 |
| Kuwait | 1990-1999 | 0 | 2 | 2 |
| Kuwait | 2000-2009 | 2 | 4 | 4 |
| Kuwait | 2010-2018 | 2 | 4 | 4 |
| Lebanon | 2010-2018 | 19 | 45 | 45 |
| Oman | 2000-2009 | 28 | 31 | 34 |
| Oman | 2010-2018 | 8 | 35 | 42 |
| Palestine | 2000-2009 | 0 | 6 | 6 |
| Palestine | 2010-2018 | 0 | 8 | 8 |
| Qatar | 1990-1999 | 11 | 13 | 13 |
| Qatar | 2000-2009 | 3 | 6 | 6 |
| Qatar | 2010-2018 | 1 | 5 | 5 |
| Saudi Arabia | 1990-1999 | 195 | 272 | 272 |
| Saudi Arabia | 2000-2009 | 289 | 436 | 436 |
| Saudi Arabia | 2010-2018 | 273 | 446 | 446 |
| Syrian Arab Republic | 2010-2018 | 0 | 75 | 75 |
| Turkey | 2000-2009 | 57 | 155 | 155 |
| Turkey | 2010-2018 | 79 | 199 | 199 |
| India | 2000-2009 | 18 | 197 | 197 |
| India | 2010-2018 | 72 | 199 | 199 |
| China | 1990-1999 | 0 | 14 | 14 |
| China | 2000-2009 | 607 | 776 | 802 |
| China | 2010-2018 | 5283 | 6522 | 6597 |
| Taiwan (Province of China) | 1990-1999 | 41 | 56 | 56 |
| Taiwan (Province of China) | 2000-2009 | 94 | 119 | 119 |
| Taiwan (Province of China) | 2010-2018 | 149 | 194 | 194 |
| American Samoa | 1990-1999 | 0 | 1 | 1 |
| American Samoa | 2000-2009 | 0 | 2 | 2 |
| American Samoa | 2010-2018 | 0 | 2 | 2 |
| Fiji | 2000-2009 | 3 | 49 | 49 |
| Fiji | 2010-2018 | 2 | 27 | 27 |
| Guam | 1990-1999 | 5 | 6 | 6 |
| Guam | 2000-2009 | 3 | 7 | 7 |
| Guam | 2010-2018 | 3 | 8 | 8 |
| Kiribati | 1990-1999 | 0 | 12 | 12 |
| Kiribati | 2000-2009 | 0 | 9 | 9 |
| Northern Mariana Islands | 1990-1999 | 0 | 1 | 1 |
| Northern Mariana Islands | 2000-2009 | 1 | 2 | 2 |
| Northern Mariana Islands | 2010-2018 | 0 | 2 | 2 |
| Palau | 2010-2018 | 0 | 3 | 3 |
| Malaysia | 2000-2009 | 647 | 1437 | 2838 |
| Malaysia | 2010-2018 | 1060 | 1711 | 2397 |
| Maldives | 2010-2018 | 0 | 4 | 4 |
| Mauritius | 1990-1999 | 0 | 3 | 3 |
| Mauritius | 2000-2009 | 19 | 29 | 29 |
| Mauritius | 2010-2018 | 97 | 106 | 106 |
| Philippines | 2000-2009 | 27 | 865 | 5671 |
| Philippines | 2010-2018 | 205 | 1217 | 5177 |
| Sri Lanka | 2000-2009 | 1 | 124 | 124 |
| Sri Lanka | 2010-2018 | 2 | 118 | 118 |
| Thailand | 2010-2018 | 4885 | 7184 | 14390 |
| South Africa | 1990-1999 | 8179 | 54327 | 85124 |
| South Africa | 2000-2009 | 13229 | 119727 | 211885 |
| South Africa | 2010-2018 | 21010 | 88661 | 146837 |
| Zimbabwe | 1990-1999 | 0 | 20430 | 27295 |
| Zimbabwe | 2000-2009 | 23362 | 29506 | 33463 |
| Cabo Verde | 2010-2018 | 74 | 94 | 94 |
| Ghana | 2000-2009 | 8594 | 14118 | 16774 |

| Appendix Table 3. ICD9 Garbage Package ICD Codes | |
| --- | --- |
| **Garbage Package** | **ICD codes** |
| All, Ill Defined code for causes of death | 076-078.2, 110-111.9, 125-125.3, 126-127, 127.2-127.9, 131-133, 133.8-134.9,  139, 139.1, 139.9, 247-248, 264-264.9, 274-274.9, 289, 289.1-289.3, 293, 294-  294.0, 296-302.9, 306-307.0, 307.2-307.4, 307.6-319.9, 327-327.1, 328-329, 338-  339.1, 339.3-339.8, 346-346.9, 350-353.6, 354-355.9, 360-362, 362.1-376, 376.2-  380.9, 384-389.9, 520-529.9, 536, 536.3, 536.8-537, 537.7, 537.9, 564, 564.8-  564.9, 603-603.9, 605-608.1, 608.3-609, 611-612.1, 615-616.9, 621-621.3, 622-  622.0, 622.8-623.6, 623.8-624.5, 624.8-629, 629.9, 690-693.9, 695, 695.8-706.9,  708-709.9, 712-713.8, 715-716, 716.2-721.6, 721.8-730, 730.3, 731-731.9, 733,  733.2-734.2, 737-738, 738.2-739.9, 780, 780.1, 780.4-780.5, 780.7-782.3, 782.6-  783.1, 783.3-784.6, 784.9, 786, 786.6, 786.8, 787, 787.3-788, 788.3-789, 790-  790.1, 790.4-796.1, 796.3-796.9, 798, 798.1-799, 799.2-799.3, 799.5-799.9 |
| Senility | 797-797.9 |
| Unspecified Infectious Diseases | 000-000.9, 030-030.9, 067-069, 078, 078.8-079, 079.8-079.9, 089-089.9, 105-  109.9, 119, 136, 136.8-136.9, 139, 139.8 |
| Sepsis (Non- maternal and neonatal sepsis) | 038-038.9, 040-040.0, 041, 041.1, 286, 286.6, 780, 780.6, 785, 785.4 |
| Pneumonitis | 507-507.9, 513-513.1 |
| Intermediate cause for CNS | 293-293.9, 331, 331.3-331.4, 332, 332.1-332.9, 347-349, 349.9 |
| Pulmonary Embolism | 415-415.9 |
| Unspecified Hemorrhage | 459-459.0 |
| Acute Respiratory Failure | 514-515.9, 518-518.0, 518.4-518.5 |
| Chronic respiratory failure | 518, 518.8 |
| Osteomyelitis | 730-730.0, 730.2, 730.7-730.9 |
| Cachexia | 783, 783.2, 799, 799.4 |

| Anemia Unspecified | 280-281, 285-285.9 |
| --- | --- |
| Hepatic Failure | 570-570.9, 573, 573.1-573.3, 782, 782.4, 789, 789.1-789.2, 789.5 |
| Acute kidney failure | 584-584.9, 586-587.9 |
| Peritonitis & Acute Abdomen | 567-568.9, 789-789.0, 789.3-789.4, 789.6-789.9 |
| Pleurisy, Pyothorax | 510-511.9, 518, 518.3 |
| Fluid, Electrolyte, Acid Base Disorders | 276-276.9 |
| Shock, Cardiac Arrest, Coma | 427, 427.5, 427.9, 458-458.9, 780-780.0, 780.2-780.3, 785, 785.5-785.9, 799-799.1 |

| Appendix Table 4. ICD10 Garbage Package ICD Codes | |
| --- | --- |
| **Garbage Package** | **ICD codes** |
| All, Ill Defined code for causes of death | A59-A59.9, A71-A71.9, A74-A74.0, B07-B07.9, B30-B30.9, B35-B36.9, B85- B85.4, B87-B88.9, B94-B94.0, D68, E15-E16, E50-E50.9, E64, E64.1, F06, F06.3-F06.4, F07, F07.2, F09-F09.9, F30-F49, F51-F99.0, G32-G32.8, G43- G44.2, G44.4-G44.8, G47-G47.2, G47.4-G47.9, G50-G60.9, G62-G62.0, G62.2-  G65.2, G89-G89.4, G99-H05, H05.2-H69.9, H71-H99, K00-K19, K30, L20-L30.9, L40-L50.9, L52-L54.8, L56-L56.2, L56.4-L56.5, L57-L57.9, L59-L68.9, L70- L76.8, L80-L87.9, L90-L92.9, L94-L96, L98, L98.5-L99.8, M04, M10-M12.0, M12.2-M29, M37-M39, M43, M43.2-M49, M49.2-M65, M65.1-M71, M71.2- M72.4, M72.8-M73, M73.8-M79.9, M83-M85.9, M87, M87.2-M87.9, M89,  M89.1-M89.4, M90-M99.9, N32, N32.8-N33.8, N35-N35.9, N37-N37.8, N39,  N39.3-N39.8, N42-N44, N44.1-N44.8, N46-N48.9, N50-N53.9, N61-N64.9, N91-  N91.5, N95, N95.1-N95.9, N97-N97.9, R07-R07.0, R08-R09, R09.3, R12-R12.0,  R14-R15.9, R19-R19.6, R19.8-R23, R23.1-R30.9, R32-R39.9, R41-R49.9, R51-  R53.8, R55-R55.0, R58-R63.3, R63.5, R63.8-R65.1, R66-R72.9, R74-R78, R78.6- R94.8, R96-R99.9, U05, U08-U81, U89-U99, Z00-Z15.8, Z17-ZB0 |
| Senility | R54-R54.9 |
| Unspecified Infectious Diseases | A14-A14.9, A29-A30.9, A45-A45.9, A47, A61-A62, A72-A73, A76, A97, B11- B14, B28-B29, B31-B32.4, B61-B62, B68-B68.9, B73-B74.2, B76-B76.9, B78- B81.8, B84, B92-B94, B94.8-B95, B95.6-B97.1, B97.3, B97.7-B99.9 |
| Unspecified Viral Diseases | B08-B09, B34-B34.1, B34.3-B34.9, G93, G93.3 |
| Pulmonary Embolism | I26-I26.9 |
| Sepsis (Non- maternal and neonatal sepsis) | A40-A41.9, A48-A48.0, A48.3, A49-A49.1, D65-D65.9, I76, R02-R02.9, R50- R50.1, R50.8-R50.9, R56-R56.0, R65, R65.2 |
| Pneumonitis | J69-J69.9, J85-J85.3 |
| Chronic respiratory failure | J96, J96.1-J96.8 |
| Osteomyelitis | M86-M86.2, M86.5-M86.9 |
| Acute kidney failure | N17, N19-N19.9 |
| Anemia Unspecified | D50-D50.0, D50.9, D62-D63, D63.8-D64, D64.1-D64.9, D69, D69.9 |

| Hepatic Failure | K71-K71.6, K71.8-K72.9, R16-R18.9 |
| --- | --- |
| Fluid, Electrolyte, Acid Base Disorders | E86-E87.6, E87.8-E87.9 |
| Intermediate cause for CNS | G91-G91.2, G91.4-G93, G93.1-G93.2, G93.4-G93.6, G94-G94.8 |
| Cachexia | R63, R63.4, R63.6, R64 |
| Peritonitis & Acute Abdomen | K65-K66.1, K66.9, K68-K68.9, R10-R10.9 |
| Acute Respiratory Failure | J80-J81.0, J96-J96.0, J96.9, J98, J98.1-J98.3 |
| Shock, Cardiac Arrest, Coma | I46-I46.9, I95-I95.1, I95.8-I95.9, R03, R03.1, R09-R09.0, R09.2, R09.8, R40- R40.4, R55, R56, R56.1-R57.9 |
| Pleurisy, Pyothorax | J86-J86.9, J90-J90.0, J94-J94.1, J94.8-J94.9, R09, R09.1 |

| Appendix Table 5. Misclassified HIV deaths, ICD 9 codes | |
| --- | --- |
| **GBD cause name** | **ICD codes** |
| Paratyphoid fever | 002.1-002.9 |
| Other diarrheal diseases | 007.8, 008.3-009.9 |
| Respiratory tuberculosis | 010-012.9 |
| Tuberculosis of nervous system | 013-013.9, 320.4 |
| Tuberculosis of intestines, peritoneum and mesenteric glands | 014-014.9 |
| Tuberculosis of bones and joints | 015-015.9 |
| Tuberculosis of genitourinary system | 016-016.9 |
| Tuberculosis of other organs | 017-017.1, 017.3-019.9 |
| Tuberculous peripheral lymphadenopathy | 017.2 |
| Other unspecified infectious diseases | 034, 034.1-034.9, 040, 040.1-041.0, 046-  046.9, 050-051.9, 057-059.9, 074-075.9,  078.4-078.7, 079-079.5, 079.7, 101-101.6,  104-104.9, 136-136.2, 139, 323.0-323.3,  390-390.9, 392, 392.9, 771.0-771.2 |
| HIV/AIDS resulting in other diseases | 042-044.9 |
| Encephalitis | 062-064.9, 139.0, 323, 323.4-323.9 |
| Other neglected tropical diseases | 065-066.9, 080-083.9, 087-088, 088.8-  088.9, 122.5-122.7, 123-123.0, 123.2-  124.9, 125.4-125.6, 125.9, 127, 127.1,  128-129.0 |

| Malaria | 084-084.9 |
| --- | --- |
| Other sexually transmitted infections | 099-099.9 |
| Drug-susceptible tuberculosis | 137-137.9, 138.0-138.9, 730.4-730.6 |
| Cervical cancer | 180-180.9, 219.0, 233.1, 622.1-622.2,  622.7 |
| Hyperthyroidism | 242-242.9, 775.3 |
| Other nutritional deficiencies | 244.2, 265-269.9, 281.0-281.9, 716.0 |
| Other endocrine, metabolic, blood, and immune disorders | 246-246.9, 251-251.2, 251.4-253.6, 253.8-  256.3, 256.8-259.1, 259.3-259.9, 270-  271.9, 273-273.9, 275-276, 277, 277.1-  277.2, 277.4-277.9, 278.2-278.8, 286-  286.5, 286.7-289.0, 289.4-289.7 |
| Other neurological disorders | 330-330.9, 331.5-331.9, 333-334.9, 335.3,  336-337.9, 341-341.9, 349, 349.2-349.8,  353.8-353.9, 356-356.9, 357.0-357.1,  357.3-357.4, 357.7, 358-359.9, 775.2 |
| Multiple sclerosis | 340-340.9 |
| Idiopathic epilepsy | 345-345.9 |
| Other lower respiratory infections | 466-469, 470.0, 480-480.0, 480.2-480.9,  482-482.1, 482.3-482.8, 483.0-483.9,  484.1-484.2, 484.6-484.7 |
| Pneumococcal pneumonia | 481-481.9 |
| H influenzae type B pneumonia | 482.2 |
| Influenza | 487-489 |
| Diarrheal diseases | 558.2-558.9 |

Pancreatitis

577-577.9, 579.4

| Appendix Table 6. Misclassified HIV deaths, ICD10 codes | |
| --- | --- |
| **GBD cause name** | **ICD codes** |
| Paratyphoid fever | A01.1-A01.4 |
| Other diarrheal diseases | A04, A04.6, A04.8-A04.9, A07, A07.3-A07.4, A08, A08.3-A09.9, K52.1-K52.3, R19.7 |
| Respiratory tuberculosis | A10-A14, A15-A16.9 |
| Tuberculosis of nervous system | A17-A17.9 |
| Tuberculosis of other organs | A18, A18.4-A19.9, K67.3 |
| Tuberculosis of bones and joints | A18.0 |
| Tuberculosis of genitourinary system | A18.1 |
| Tuberculous peripheral lymphadenopathy | A18.2 |
| Tuberculosis of intestines, peritoneum and mesenteric glands | A18.3 |
| Other unspecified infectious diseases | A32-A32.9, A38-A38.9, A48.2, A48.4-A48.5, A65-A65.0, A69- A69.1, A74, A74.8-A74.9, A81-A81.9, A88-A89.9, B03-B04, B25-B25.9, B27-B27.9, B29.4, B33, B33.3-B33.8, B47-B48.8,  B91, B95-B95.5, D70.3, D89.3, F02.1, G14-G14.6, I00, I02, I02.9,  I98.1, K67.8, K75.3, K76.3, K77.0, M89.6, P35, P35.1-P35.2, P35.9, P37, P37.2, P37.5-P37.9 |
| Other lower respiratory infections | A48.1, A70, B34.2, B97.2, J12-J12.0, J12.2-J12.9, J15-J15.2,  J15.5, J15.7-J15.8, J16-J16.9, J20-J21.9, J91.0, P23.0-P23.4 |
| Other sexually transmitted infections | A57-A58, A63-A63.8, B63 |

| Other neglected tropical diseases | A68-A68.9, A69.2-A69.9, A75-A75.9, A77-A79.9, A92-A94.0,  A96-A96.9, A98-A98.3, A98.5-A98.8, B33.0-B33.1, B60-B60.8, B67.5-B67.7, B70-B71.9, B74.3-B75, B83-B83.8, P37.1 |
| --- | --- |
| Encephalitis | A83-A86.4, B94.1, F07.1, G04-G05.8, G21.3 |
| HIV/AIDS resulting in other diseases | B20, B20.1-B24.9, F02.4 |
| HIV/AIDS - Drug-susceptible Tuberculosis | B20.0 |
| Malaria | B50-B53.8 |
| Drug-susceptible tuberculosis | B90-B90.9, K93.0, M49.0, N74.1, P37.0 |
| Cervical cancer | C53-C53.9, D06-D06.9, D26.0 |
| Other nutritional deficiencies | D51-D52.0, D52.8-D53.9, E00-E02, E51-E61.9, E63-E64, E64.2- E64.9, M12.1 |
| Other endocrine, metabolic, blood, and immune disorders | D66-D67, D68.0-D69.4, D69.6-D69.8, D70-D70.0, D70.4-D75.8,  D76-D77, D86.8, D89-D89.2, E07.0, E16.1-E16.9, E20-E23.0,  E23.2-E24.1, E24.3, E24.8-E27.2, E27.4-E28.1, E28.3-E34, E34.1-E34.8, E67-E68, E70-E77.9, E79-E83.9, E85-E85.2, E88- E88.2, E88.4-E88.9 |
| Hyperthyroidism | E05-E05.9 |
| Other neurological disorders | F02.2, G10-G12.1, G13-G13.8, G23-G24, G24.1-G25.0, G25.2-  G25.3, G25.5, G25.8-G26.0, G36-G37.9, G61-G61.9, G70-G71.1, G71.3-G71.9, G73-G73.7, G90-G90.9, G95-G95.9 |
| Multiple sclerosis | G35-G35.9 |
| Idiopathic epilepsy | G40-G41.9 |
| Influenza | J09-J11.8, U04-U04.9 |

| Pneumococcal pneumonia | J13-J13.9, J15.3-J15.4, J15.6 |
| --- | --- |
| H influenzae type B pneumonia | J14-J14.0 |
| Pancreatitis | K85-K86.9 |

## Appendix Table 7. Covariates used in multiple causes of death analysis

| **Intermediate cause** | **Covariates** |
| --- | --- |
| Acute renal failure; acute respiratory failure; cachexia; empyema; unspecified central nervous system disorders; osteomyelitis; peritonitis; pneumonitis; pulmonary embolism; shock, cardiac arrest, and coma^a^ | Healthcare access and quality index^1^; sex;  age group; underlying cause |
| Chronic respiratory failure | Healthcare access and quality index^1^;  smoking prevalence (age-standardized)^2^; sex; age group; underlying cause |
| Fluid, electrolyte, and acid-base disorders | Healthcare access and quality index^1^; summary exposure value for unsafe sanitation^2^; sex;  age group; underlying cause |
| Hepatic failure | Healthcare access and quality index^1^;  alcohol binge drinker proportion (age-standardized)^2^; hepatitis B seroprevalence (age standardized)^3^; sex; age group; underlying cause |
| Sepsis^b^ (excluding maternal and neonatal sepsis) | Healthcare access and quality index^1^; sex; underlying cause |

^a^ Underlying cause was included as a fixed effect for “shock, cardiac arrest, and coma”. For all other **intermediate** causes, the underlying cause was included as a random effect.

^b^Individual models were run for each age group.

## Appendix Table 8. Redistribution Proportions for Pulmonary Embolism, All Ages Both Sexes, Year 2015, Top 20 Underlying Causes

| **Cause** | **Central Europe, Eastern Europe, and Central Asia** | **High-income** | **Latin America and Caribbean** | **North Africa and Middle East** | **South Asia** | **Southeast Asia, East Asia, and Oceania** | **Sub-Saharan Africa** |
| --- | --- | --- | --- | --- | --- | --- | --- |
| HIV/AIDS | 0.30% | 0.06% | 0.63% | 0.14% | 0.35% | 0.28% | 7.89% |
| Tuberculosis | 0.33% | 0.06% | 0.35% | 0.36% | 3.63% | 1.00% | 5.55% |
| Lower respiratory infections | 1.72% | 3.05% | 4.24% | 2.31% | 3.40% | 2.26% | 7.52% |
| Esophageal cancer | 0.53% | 0.82% | 0.65% | 0.38% | 0.65% | 2.15% | 0.90% |
| Stomach cancer | 2.60% | 2.18% | 2.93% | 2.00% | 1.61% | 5.04% | 1.28% |
| Colon and rectum cancer | 4.02% | 4.92% | 2.79% | 1.93% | 1.40% | 3.60% | 1.25% |
| Pancreatic cancer | 2.36% | 3.87% | 1.90% | 1.33% | 0.74% | 1.87% | 0.77% |
| Tracheal, bronchus, and lung cancer | 5.58% | 8.49% | 3.49% | 3.44% | 1.69% | 8.80% | 1.53% |
| Breast cancer | 2.33% | 2.86% | 2.29% | 1.95% | 2.08% | 1.94% | 2.00% |
| Ovarian cancer | 1.40% | 1.53% | 1.10% | 0.67% | 0.82% | 0.84% | 0.59% |
| Prostate cancer | 1.24% | 2.16% | 2.37% | 0.84% | 0.56% | 0.79% | 1.66% |
| Ischemic heart disease | 20.26% | 7.98% | 8.62% | 16.53% | 11.82% | 10.47% | 5.48% |
| Ischemic stroke | 9.60% | 3.48% | 3.42% | 5.86% | 3.00% | 7.41% | 2.59% |
| Intracerebral hemorrhage | 2.69% | 1.32% | 2.03% | 2.08% | 3.81% | 7.38% | 4.10% |
| Hypertensive heart disease | 1.61% | 1.41% | 1.67% | 3.22% | 1.32% | 2.68% | 2.45% |
| Other cardiovascular and circulatory diseases (internal) | 14.69% | 15.16% | 15.12% | 25.59% | 14.50% | 6.35% | 17.70% |
| Chronic obstructive pulmonary disease | 2.32% | 4.52% | 4.30% | 3.09% | 11.16% | 8.98% | 2.53% |
| Diabetes mellitus type 2 | 0.98% | 1.28% | 3.99% | 2.21% | 2.34% | 2.03% | 2.60% |
| Chronic kidney disease | 0.56% | 1.59% | 3.25% | 2.18% | 1.77% | 1.55% | 1.58% |
| Falls | 3.73% | 6.25% | 5.57% | 3.51% | 10.68% | 6.27% | 3.04% |

**References**

1. Fullman N, Yearwood J, Abay SM, Abbafati C, Abd-Allah F, Abdela J, et al. Measuring performance on the Healthcare Access and Quality Index for 195 countries and territories and selected subnational locations: a systematic analysis from the Global Burden of Disease Study 2016. The Lancet. 2018;391(10136):2236-71.
2. Murray CJ, Aravkin AY, Zheng P, Abbafati C, Abbas KM, Abbasi-Kangevari M, et al. Global burden of 87 risk factors in 204 countries and territories, 1990–2019: a systematic analysis for the Global Burden of Disease Study 2019. The Lancet. 2020;396(10258):1223-49.
3. Vos T, Lim SS, Abbafati C, Abbas KM, Abbasi M, Abbasifard M, et al. Global burden of 369 diseases and injuries in 204 countries and territories, 1990–2019: a systematic analysis for the Global Burden of Disease Study 2019. The Lancet. 2020;396(10258):1204-22.

## Appendix Figure 1. GBD Regions and Super Regions


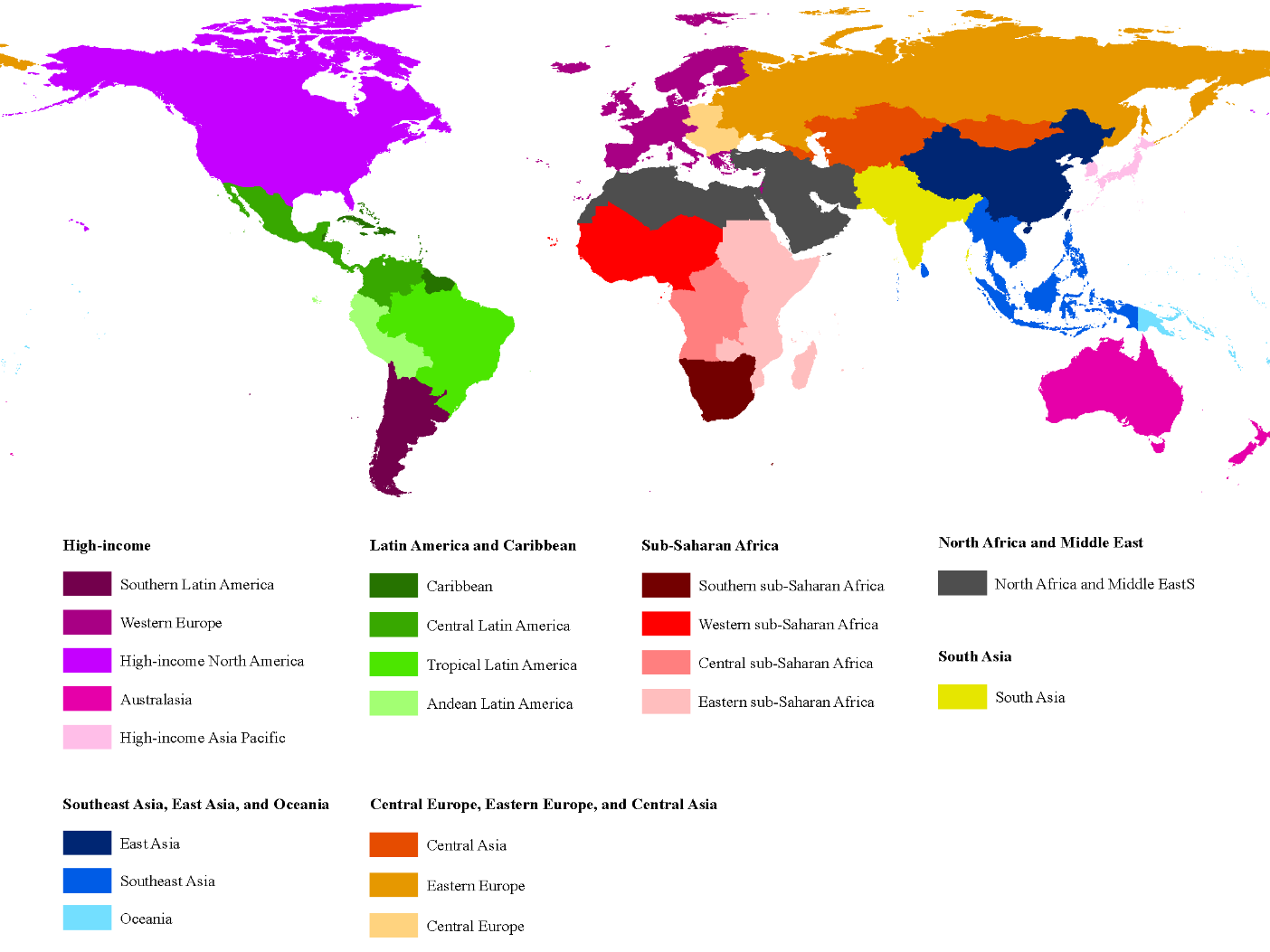


## Appendix Figure 2. Overall causes-of-death data quality by country


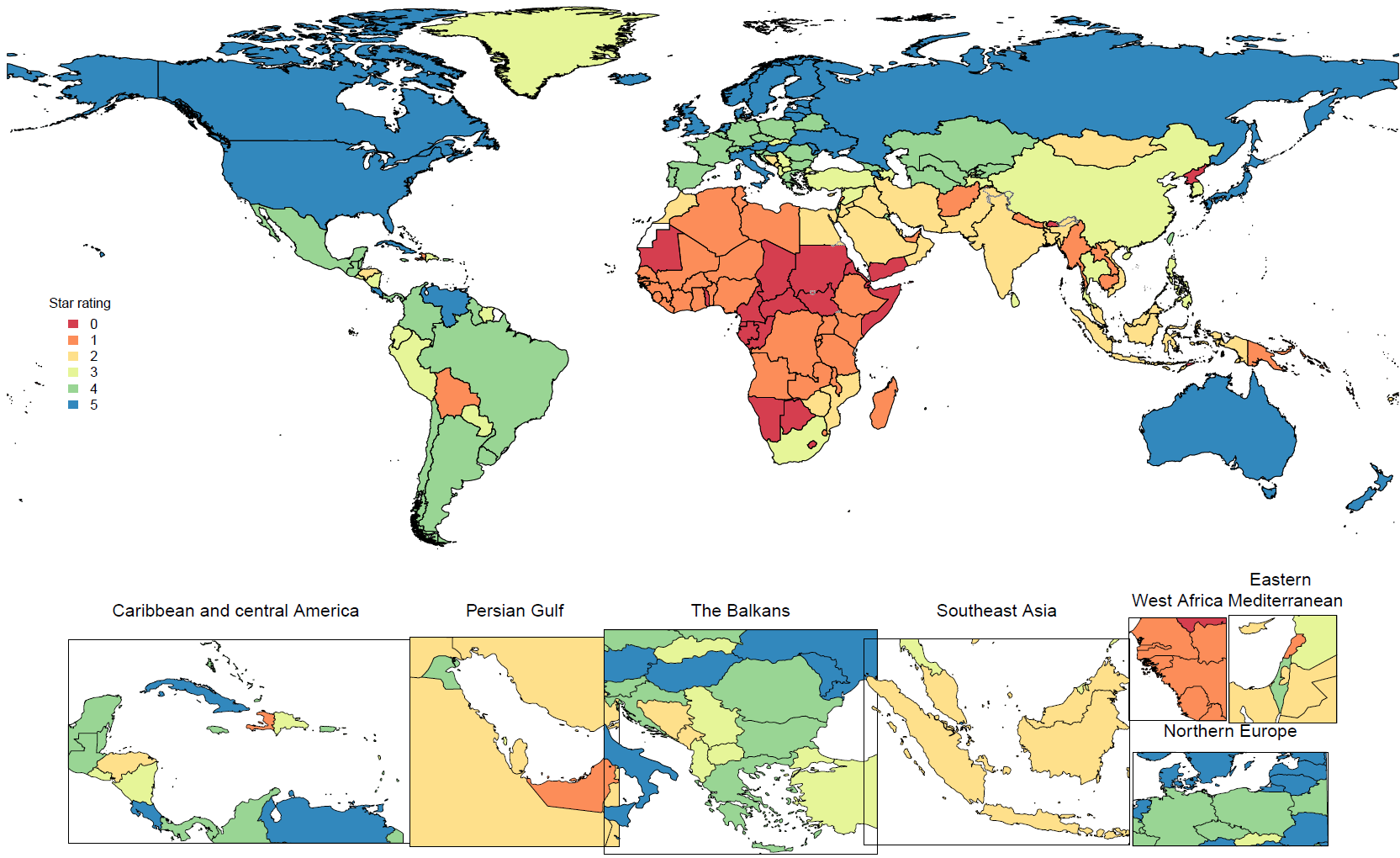


## Appendix Figure 3. Variation in misclassification over time by super region.


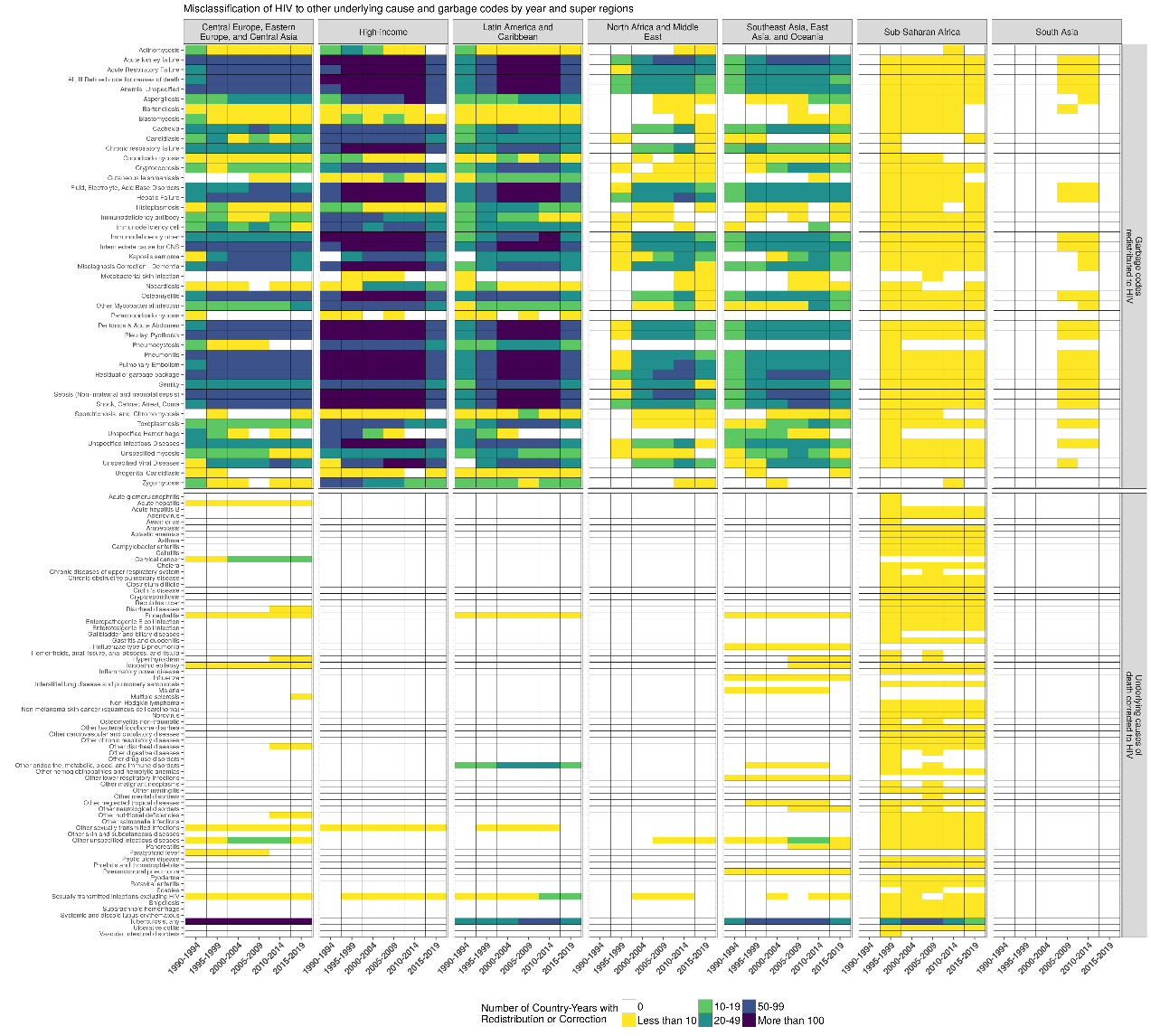


## Estimating redistribution uncertainty

While redistribution uncertainty was not used to inform the results presented in this paper, uncertainty was incorporated into the GBD cause of death modeling.

To calculate the uncertainty in the garbage code redistribution algorithms, we first computed percent garbage by location, year, age, sex, and cause, using the following equation

$$pct_{garbage}=\frac{deaths_{redistributed}-deaths_{raw}}{deaths_{redistributed}}$$

We then ran a mixed-effect linear regression model to predict the logit percent garbage as a function of age-standardized relative rate of major garbage codes.

$$logit\left( pct_{garbage_{ij}} \right)= \beta_{0}+\beta_{1}*\log\left( R{R_{major garbage}}_{ij} \right)+\beta_{2}*age_{ij}+\gamma_{1j}*\log\left( RR_{major garbage_{ij}} \right)+u_{j}+e_{ij}, \theta_{\left\{ i \right\}}\sim N(0,\sigma^{2})$$

Where: $i$ denotes dataset-location-year-age-sex-cause data points nested within $j$ groups by GBD region, and

$RR_{majorgarbage}$ is the age-standardised relative rate of major garbage computed as

$$RR_{major garbage}=\frac{AS{MR}_{major garbage}}{ASMR_{all deaths}}$$

Next, the residual variance, as estimated by the mean absolute deviation, for each cause, sex, and age group, was used to calculate uncertainty around each data point.
